# Phylogenetic Analysis and DNA-based Species Confirmation in *Anopheles* (*Nyssorhynchus*)

**DOI:** 10.1371/journal.pone.0054063

**Published:** 2013-02-04

**Authors:** Peter G. Foster, Eduardo S. Bergo, Brian P. Bourke, Tatiane M. P. Oliveira, Sandra S. Nagaki, Denise C. Sant’Ana, Maria Anice M. Sallum

**Affiliations:** 1 Department of Life Sciences, Natural History Museum, London, United Kingdom; 2 Superintendência de Controle de Endemias, Secretaria de Estado da Saúde de São Paulo, Araraquara, São Paulo, Brazil; 3 Departamento de Epidemiologia, Faculdade de Saúde Pública, Universidade de São Paulo, São Paulo, Brazil; Centro de Pesquisas René Rachou, Brazil

## Abstract

Specimens of neotropical *Anopheles* (*Nyssorhynchus*) were collected and identified morphologically. We amplified three genes for phylogenetic analysis–the single copy nuclear *white* and *CAD* genes, and the *COI* barcode region. Since we had multiple specimens for most species we were able to test how well the single or combined genes were able to corroborate morphologically defined species by placing the species into exclusive groups. We found that single genes, including the *COI* barcode region, were poor at confirming species, but that the three genes combined were able to do so much better. This has implications for species identification, species delimitation, and species discovery, and we caution that single genes are not enough. Higher level groupings were partially resolved with some well-supported groupings, whereas others were found to be either polyphyletic or paraphyletic. There were examples of known groups, such as the Myzorhynchella Section, which were poorly supported with single genes but were well supported with combined genes. From this we can infer that more sequence data will be needed in order to show more higher-level groupings with good support. We got unambiguously good support (0.94–1.0 Bayesian posterior probability) from all DNA-based analyses for a grouping of *An. dunhami* with *An. nuneztovari* and *An. goeldii*, and because of this and because of morphological similarities we propose that *An. dunhami* be included in the Nuneztovari Complex. We obtained phylogenetic corroboration for new species which had been recognised by morphological differences; these will need to be formally described and named.

## Introduction

Malaria is among the world’s most important infectious diseases. There were approximately 216 million cases of malaria reported in 2011 and an estimated 655,000 deaths in 2010 [1]. The disease is a major obstacle to social and economic development in affected countries [2], and although considerable funding has helped decrease the incidence of malaria by an estimated 17% since 2000, the failure to maintain effective malaria control strategies can lead to resurgence in historically endemic regions [1], [3]. Climate change [4] and deforestation [5] are also likely to play an important role in the appearance of malaria in non-endemic and newly inhabited regions, respectively. Effective approaches to species identification are essential in vector incrimination, the assessment of malaria risk and development of malaria control strategies [6]. The focus of such work centres on the genus *Anopheles*, which contains all known vectors of malaria, with many forming cryptic species complexes [7]. Such species are morphologically indistinguishable and for many of the most important vectors of malaria a molecular approach is the only effective tool for resolving species and species relationships [8].

There are nine dominant *Anopheles* vector species in the Americas, three species belong to the subgenus *Anopheles* and six to the *Nyssorhynchus* [9]. Among species of the subgenus *Nyssorhynchus*, *Anopheles darlingi* is the primary vector in areas of the Amazon Region [9], [10]. In addition, *An. albimanus*, species of the *An. albitarsis* Complex, *An. aquasalis*, *An. marajoara*, and *An. nuneztovari* are also dominant vectors of human *Plasmodium* [9]. Other species of the *Nyssorhynchus* may be secondary, local vectors, or were found naturally infected with *Plasmodium* sporozoites, for example, *An. benarrochi* [11], *An. rangeli* [12], [13], *An. oswaldoi* [12], [14], *An. oswaldoi* B [13], *An. strodei* [15], *An. rondoni* [16], *An. trinkae* [12], and *An. triannulatus* [17].

The Anophelinae subfamily includes 465 formally recognized species and 50 unnamed members of species complexes [7], which are subdivided into three genera – *Anopheles*, *Bironella*, and *Chagasia*. Species of the genus *Anopheles* are subdivided into seven subgenera: *Anopheles*, *Baimaia*, *Cellia*, *Kerteszia*, *Lophopodomyia*, *Nyssorhynchus*, and *Stethomyia*. Worldwide, the primary vectors of human malaria parasites belong to the subgenera *Anopheles*, *Cellia* and *Nyssorhynchus* [18]. Most phylogenetic and phylogeographic studies regarding Anophelinae involved medically important species or species groups. The most comprehensive phylogenetic study, in terms of the number of species sampled, is that of Sallum *et al*. (2000) [19], which employed morphological characters. Results of phylogenetic analysis, using either morphological characters [19] or sequences of mitochondrial and nuclear genes [20] corroborated the monophyly of Anophelinae and of subgenera *Cellia*, *Kerteszia* and *Nyssorhynchus*.

The subgenus *Nyssorhynchus* has a Neotropical distribution, with *An. albimanus* extending to the southern Neartic Region. The *Nyssorhynchus* includes 39 formally named species [21] with a current listing of 44 [22], which are subdivided between the Albimanus Section (Faran 1980) [23], Argyritarsis Section (Linthicum 1988) [24], and Myzorhynchella Section (Galvão 1941) [25]. In the Argyritarsis and Albimanus Sections there are species that were not formally validated and therefore were designated by letters added to the name of the taxon morphologically more similar. This is so in the case of *An. nuneztovari* which includes chromosomal forms A, B, and C [26]–[28]. Using ITS2 sequence data, Sierra *et al.* (2004) [29], showed that *An. nuneztovari* B and C are conspecific, and Calado *et al.* (2008) [30] demonstrated that at least part of that which was identified as *An. nuneztovari* A may include *An. goeldii*, and so this species was resurrected from the synonymy. The Strodei Complex was named by Faran (1980) [23] and includes *An. strodei*, *An. benarrochi* and *An. rondoni*. *Anopheles strodei* contained five species in synonymy, which were described by Unti [31], [32] using characters either of the eggs or larvae. Recently, Sallum *et al.* [33] resurrected *An. albertoi* and *An. arthuri* from synonymy with *An. strodei*. The number of recognized *Nyssorhynchus* species has increased over time, and it is expected that this trend will continue. It has been proposed that *An. triannulatus* [34], *An. konderi* [35], [36], and *An. oswaldoi* [14], [36] may be more than one species under the same name. There are complexes of morphologically similar species in the Argyritarsis Section that are being revealed by genetic and phylogenetic studies using DNA sequences of nuclear and mitochondrial genes, for example five named species and four un-named lineages defined by the *COI* gene in [37]. This is also so in the *An. albitarsis* species complex which so far consists of six species [38], [39]. Additionally, the once monospecific Pictipennis Group now includes *An. atacamensis*, which was recently described from specimens collected in Atacama Desert, Chile. The Myzorhynchella Section consisted until recently of only four species; however it now includes *An. antunesi*, *An. guarani*, *An. lutzii*, *An. nigritarsis*, *An. pristinus* and *An. parvus*. *An. guarani* was revalidated by Nagaki *et al.* [40] and *An. pristinus* described by Nagaki and Sallum (in Nagaki *et al.* [41]).

Phylogenetic relationships within the subgenus *Nyssorhynchus* were investigated by Bourke *et al*. (2010) [42] employing DNA sequence data from the single copy nuclear *white* gene and the *ND6* mitochondrial gene. Generally, results of phylogenetic analyses provided support for some lineages, although the topologies for the two genes disagreed somewhat. Bayesian topology of the combined *ND6* and *white* datasets supported the Myzorhynchella Section as a natural group. Unfortunately, it was not clear if either the Argyritarsis Section or the Albimanus Section was monophyletic, and relationships within these Sections could not be fully assessed because of lack of deep resolution. Furthermore, because of the small species sample size it was not possible to assess monophyly of most groups, subgroups and complexes as defined by Faran (1980) [23] for the Albimanus Section and by Linthicum (1988) [24] for the Argyritarsis Section. However, the topology from the combined dataset confirmed monophyly of the subgroups Oswaldoi and Triannulatus of the Albimanus Section. Regarding the Argyritarsis Section, the *An. albitarsis* Complex was confirmed as a monophyletic group, and *An. lanei* as a distinct group, sister to the clade formed by species of the Albimanus and Argyritarsis Sections, without assignment to any *Nyssorhynchus* section. Inconsistencies and also lack of basal resolution obtained by Bourke *et al.* (2010) [42] may have been caused by both limited taxa sampling and poor phylogenetic information contained in the two genes employed in the analyses.

Several genes have been found to be useful in *Anopheles* systematics. The 5′ region of the *COI* gene has long been used as a DNA barcode for species identification, and also for barcoding animal life [43], [44]. The barcode has been shown to be successful for mosquito identification in Canada [45], and to both corroborate known lineages and define new lineages within the *An. albitarsis* Complex [37]. Besansky and Fahey [46] used the nuclear *white* gene to confirm the monophyly of the subfamily Anophelinae, and tribes Sabethini, Culicini and Aedini. More recently, *arginine kinase*, *CAD*, *catalase*, *enolase*, *hunchback*, and *white* nuclear protein-coding genes were used to assess genus level relationships within Culicidae and to infer divergence time for major lineages, strongly supporting the monophyly of Anophelinae and the tribes Aedini and Sabethini [47]. A larger amount of sequence data would allow broader and deeper phylogenetics, such as for example the study by Regier and Zwick [48], who used 62 protein-coding nuclear genes, using only non-synonymous change, to recover a robust higher-level phylogeny of Arthropoda.

Here we used the *COI* barcode region, and the single copy nuclear *white* and *CAD* protein-coding genes to confirm known morphologically defined species, and to estimate phylogenetic relationships and assess species complexes within the subgenus *Nyssorhynchus*. We found that species confirmation was poor using any of the three genes alone, but was good using concatenated genes. Higher level groupings were partially resolved with some well-supported groupings, whereas others were found to be either polyphyletic or paraphyletic. We propose that *An. dunhami* be included in the Nuneztovari Complex. We obtained phylogenetic corroboration for new species which had been recognised by morphological differences.

## Results and Discussion

### Species Identification and DNA-based Species Confirmation

The species and specimens used in this study are listed in Table S1. The species included many of those in the catalog by Harbach [21], and also some undefined morpho-species. These undefined taxa were identified using both male genitalia and fourth-instar larva characteristics, but since they could be differentiated from their morphologically closest relative they were marked as *sensu lato*; further studies will be necessary to name and validate these taxa. These included taxa in the Oswaldoi Subgroup which were preliminarily identified as *An. konderi s.l.* [35], [36] and *An. oswaldoi s.l.* [36], and also *An. argyritarsis s.l.* The specimens had been identified as above, and also when possible from the scanning electron microscope of the egg; the latter was used to aid identification of species of the Strodei Subgroup and Myzorhynchella Section.

Three genes were used for the molecular analysis – the single copy nuclear *white* and *CAD* genes, and the barcode region of the mitochondrial *COI* gene. The aligned concatenated genes are characterized in Table 1. Model-based phylogenetic analysis was done with two different Bayesian phylogenetic programs, MrBayes v 3.1.2 [49], and p4 [50], and for each program multiple separate “runs” were done. We have as usual pooled the results from the runs and made consensus trees from the pooled samples, but also here we have kept the results of runs separate to better show the spread that support values can take (Figures S1A–E and S2, and Tables S2, S3, and S4). Additionally, pairwise Kimura 2-parameter (K2P) distances were measured among sequences of the COI barcode region, and also of the three concatenated gene regions, and these were used to make neighbour-joining (NJ) trees (Figures S1F and S1G).

**Table 1:** Characterization of the alignment of the three gene regions.

gene	codon position	nChar	nTax	unique sequences	constant	variable	parsimony informative
concat		2298	144	143	1428	870	703
*white*		750	137	116	510	240	174
	first	250		89	204	46	25
	second	250		49	233	17	5
	third	250		114	73	177	139
*CAD*		846	129	120	440	406	332
	first	282		108	196	86	53
	second	282		102	224	58	33
	third	282		118	20	262	245
*COI*		702	144	131	478	224	194
	first	234		103	200	34	26
	second	234		47	233	1	0
	third	234		130	45	189	168

The outgroup in this analysis are the taxa *An.* (*Kerteszia*) *cruzii*, *An.* (*Anopheles*) *intermedius*, and *An.* (*Stethomyia*) *kompi*. A species of the subgenus *Kerteszia* was chosen because *Kerteszia* is a sister-group to *Nyssorhynchus*; species of the subgenera *Anopheles* and *Stethomyia* are somewhat more distant to *Nyssorhynchus* [19], [20]. Bayesian analysis with all three genes at the DNA level including all three outgroup taxa did not show any support for the outgroup (Figure S1A, Table S2B); it was only when the translations were used that support for the outgroup was seen. Even then, support for a separate outgroup was uneven, ranging from 0.0 to 0.99 Bayesian posterior probability (BPP) in different MCMC runs (Figure S2A, Table S3C). We interpreted this as indicative of unknown biases in the outgroup DNA sequences that were at least partially ameliorated in the translations. Inspection identified *An. kompi* as a difficult taxon, and it was removed from subsequent DNA-based analyses. When it was removed then the remaining two outgroup taxa generally grouped together, although not with the *COI* gene, and not fully with the *CAD* gene (Table S2B).

There were only three of the ingroup species that were represented by a single specimen; all other species in the ingroup were represented by up to ten specimens (Table S1). This allowed us to test how well the DNA-based species reflected the morphological species. We did this by asking whether in a DNA-based phylogenetic tree the specimens formed an exclusive monophyletic clade. Here we are asking whether a single gene contains sufficient phylogenetic information to place a specimen in a species, or whether this placement requires more sequence data.

**Figure 1:**
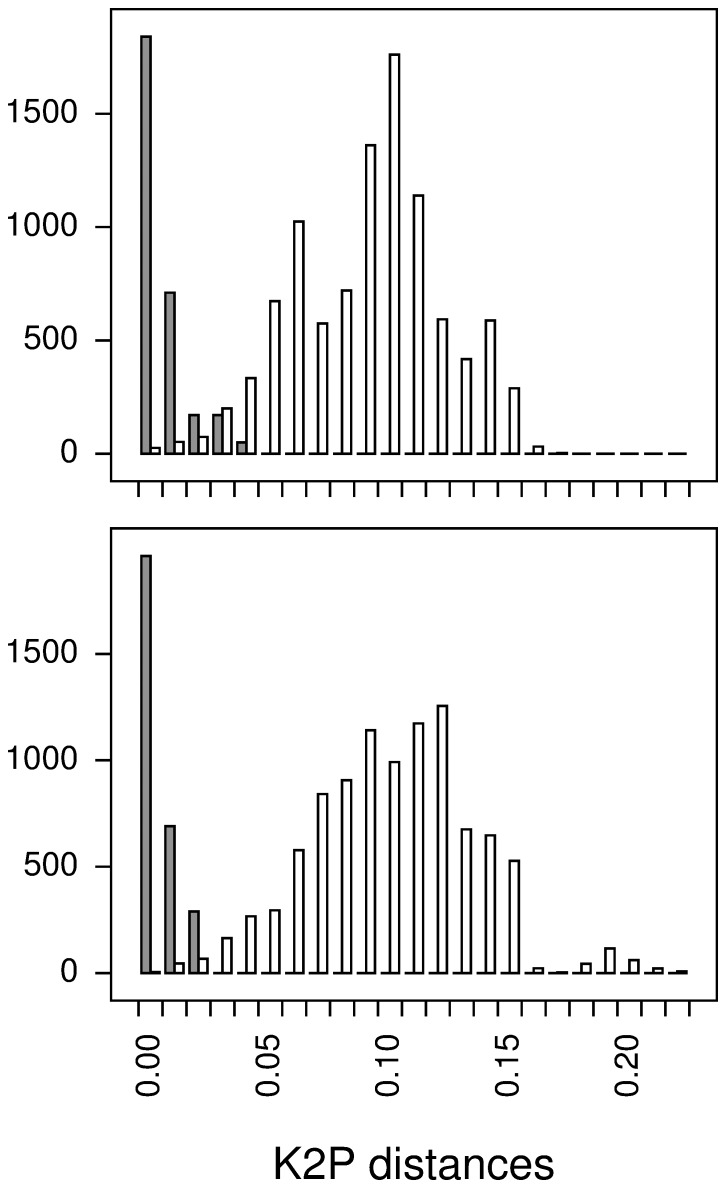
Intra- and interspecific K2P distances. The top panel shows distances between COI barcode regions, and the bottom panel shows distances between specimens using all three gene regions. Dark bars show intraspecific distances and light bars show interspecific distances. The counts of intraspecific distances have been scaled tenfold for clarity.

We obtained pairwise Kimura 2-parameter (K2P) distances from the COI barcode region, and from the three gene regions concatenated together, shown in Figure 1. For the COI barcode region, the intraspecific distances ranged from 0.0 to 0.049, while the interspecific distances ranged from 0.0015 to 0.17. For the three concatenated gene regions, the intraspecific distances ranged from 0.0 to 0.029 and the interspecific distances ranged from 0.006 to 0.225. Both the COI barcode region and the three gene region concatenation show considerable overlap in the intra- and interspecific distances, and neither has a clear barcoding gap between them. We can speculate that specimens contributing the largest intraspecific distances (Table S5) should be candidates for being considered separate species; these are discussed below. Specimen pairs with interspecific distances of less than 2% for the COI barcode region were always such that both members of the pair were from within the Oswaldoi Subgroup, within the Nuneztovari Complex, or within the Strodei Subgroup.

**Figure 2:**
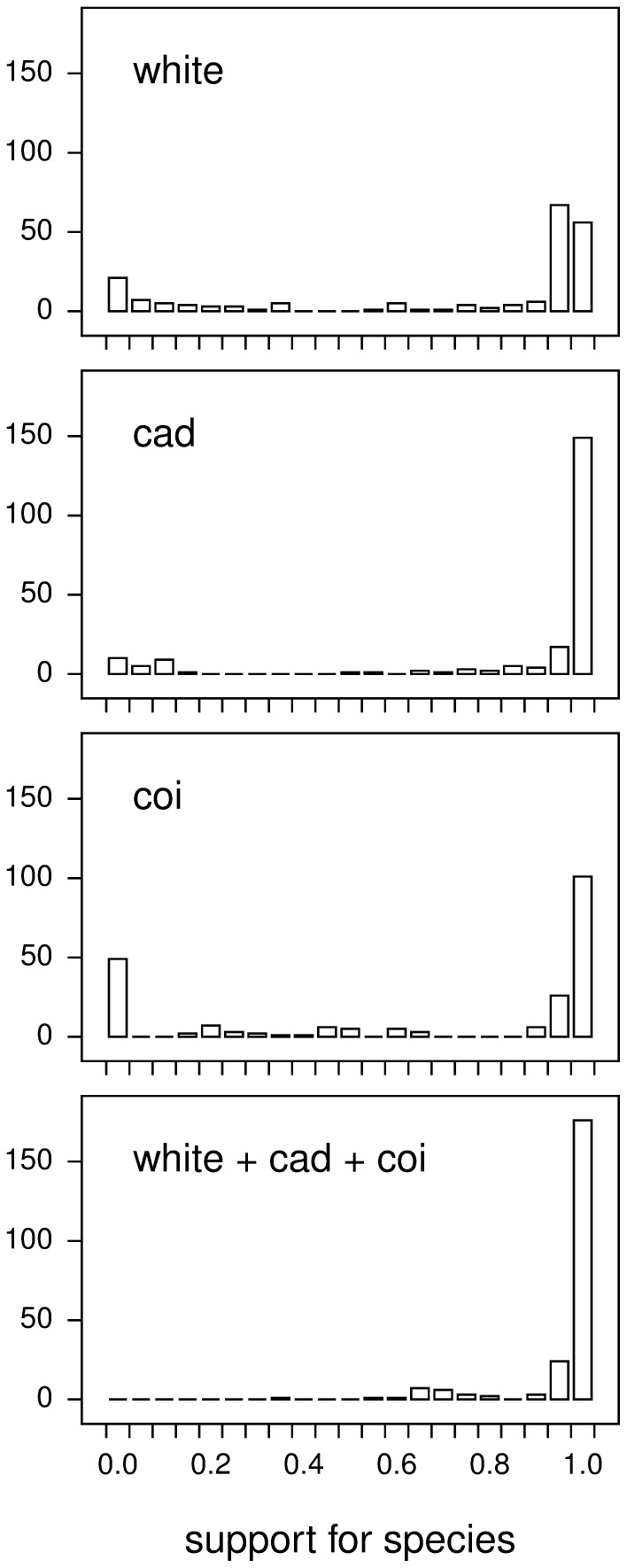
Supports for species using single genes, or using combined data from 3 genes. Bayesian posterior probabilities of species supports were taken from the posterior distributions (not the consensus trees) of the Bayesian phylogenetic analyses. The rightmost bar shows 100% support. (See Table S4 for specific values.).

In the Bayesian analyses we can ask how much clade support is given when we use a single gene (one of *white*, *CAD*, or *COI*) compared to the support for species when we use these three genes together. For those species represented by two or more specimens, we obtained support values for monophyly of the specimens for a morphologically defined species (support values were obtained from the MCMC posterior samples, as shown in Table S4, and plotted in Figure 2). The results show that using three genes combined is able to group specimens much better than single genes can do individually. All three single genes, and the *COI* gene in particular with about 23% of species having less than 10% support, are unable to show support for some species that the combined genes are able to show.

These Bayesian analyses were compared to commonly-used K2P/NJ trees (Figure S1F, S1G). Whether these trees showed monophyly for species with more than one specimen is shown in Table 2. When the COI gene was used, 8 of the species were not exclusive in the NJ tree. The COI gene results are similar to the Bayesian analysis using only the COI gene, which had several species with low BPP (Table 2, and Table S6). However, the Bayesian analysis with three genes was much better than the K2P/NJ analysis with the same set of genes (all species in the Bayesian analysis had greater than 0.69 BPP while 6 species were not exclusive in the NJ tree).

**Table 2:** Species confirmation with COI barcode and with three genes.^a^

species	COI barcode K2P/NJ^b^	COI barcode BPP^c^	three genes K2P/NJ	three genes BPP
*An. albertoi*	−	0.00	+	1.00
*An. albitarsis*	+	0.99	+	1.00
*An. antunesi*	+	0.49	−	1.00
*An. argyritarsis*	+	1.00	+	1.00
*An. arthuri*	+	0.20	+	1.00
*An. atacamensis*	+	1.00	−	1.00
*An. benarrochi*	+	1.00	+	1.00
*An. braziliensis*	+	1.00	+	1.00
*An. darlingi*	+	1.00	+	1.00
*An. deaneorum*	+	0.95	+	1.00
*An. dunhami*	+	0.27	+	0.99
*An. evansae*	−	0.00	+	1.00
*An. galvaoi*	+	1.00	+	1.00
*An. goeldii*	−	0.00	−	0.72
*An. guarani*	+	1.00	−	1.00
*An. konderi s.s.*	−	0.99	+	1.00
*An. konderi* A	+	0.50	+	0.99
*An. lanei*	+	n.a.^d^	+	1.00
*An. lutzii s.s.*	+	1.00	+	1.00
*An. lutzii* A	+	0.03	+	1.00
*An. lutzii* B	−	1.00	−	0.69
*An. marajoara*	+	0.94	+	1.00
*An. nuneztovari*	−	0.00	+	0.99
*An. oswaldoi s.s.*	−	0.00	−	0.77
*An. oswaldoi* A	+	1.00	+	1.00
*An. parvus*	+	1.00	+	1.00
*An. pristinus*	+	1.00	+	1.00
*An. rangeli*	+	1.00	+	1.00
*An. rondoni*	+	0.66	+	1.00
*An. strodei*	−	0.00	+	0.86
*An. strodei* CPform	+	1.00	+	1.00
*An. triannulatus*	+	1.00	+	1.00

a DNA-based confirmation of those morphologically-defined species with more than one specimen, using the COI barcode region only or with three genes concatenated.

b K2P distances were used to make a NJ tree. “+” if all the specimens formed an exclusive group, or “−” otherwise.

c Bayesian posterior probability, averaged from Table S6.

d Not applicable; the two COI sequences from *An. lanei* were identical, and so were homogenized for the Bayesian analysis. This can be taken as BPP of 1.00.

### Species Groups

**Figure 3:**
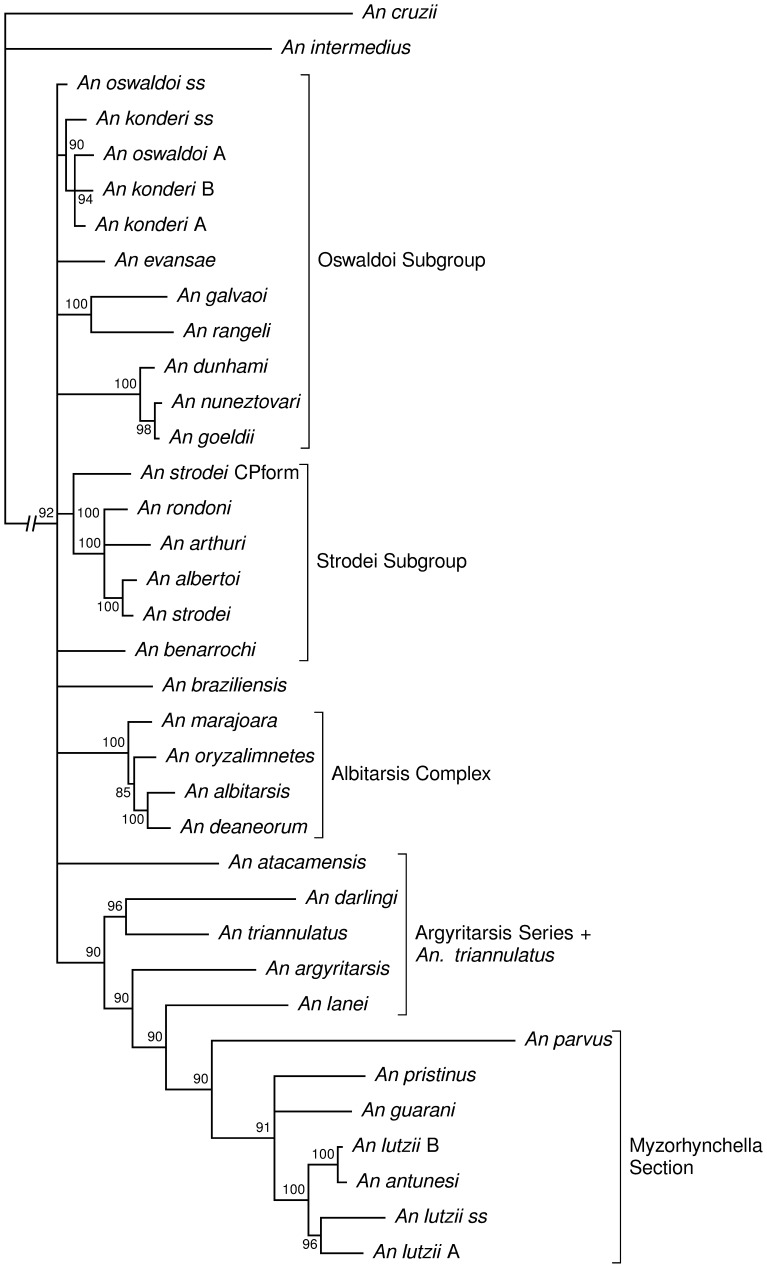
Consensus tree from a Bayesian phylogenetic analysis using all three gene regions. This is a species-level depiction of Figure S1B; see that figure for analysis details. Internal nodes show support as posterior probability, expressed as percent.

Using all three genes, the consensus tree shown in Figure 3 was obtained. The species groups supported by the analyses in this study are described with reference to Harbach [21]. *An. triannulatus* and *An. braziliensis* were found to be more or less unstable taxa in the analyses described below; for example the position of *An. triannulatus* in Figure 3 is certainly questionable. Agreement between our phylogenetic analysis and the current classification hierarchy was uneven. Of the three Sections that we sampled from (the Albimanus, Myzorhynchella, and Argyritarsis Sections), only the Myzorhynchella Section had support as a clade; it had support from analysis of translations (0.77–0.97 BPP), and support from combined genes (0.70–1.0 BPP), but not from individual genes (0.0–0.12 BPP, with a single 0.8 BPP; Tables S2 and S3). Patterns seen within these Sections are described here (note that we only sampled from the Oswaldoi Series from the Albimanus Section).

#### Oswaldoi series

The Oswaldoi Series is composed of the Oswaldoi Group and the Triannulatus Group, and in turn the Oswaldoi Group is composed of the Oswaldoi Subgroup and the Strodei Subgroup. There is little support for the Oswaldoi Subgroup; there is some, but uneven, support for this grouping from the combined DNA analysis (0.0–0.9 BPP), but not from any of the three genes individually (0.0–0.14 BPP, Table S2). Within the Oswaldoi Subgroup, there is support for various groupings from the DNA analyses (the translations do not resolve within this Complex). Harbach [21] cites the Nuneztovari Complex within the Oswaldoi Subgroup, composed of *An. goeldii* and *An. nuneztovari* [30]. We got support from most analyses for a grouping of *An. goeldii* and *An. nuneztovari* consistent with the Nuneztovari Complex (0.91–1.0 BPP using all three gene regions), but we got consistent support from all DNA analyses for this grouping including *An. dunhami* (0.94–1.0), and we suggest that *An. dunhami* should be placed in this Complex. All three species can be easily recognized as morphologically similar species by characteristics of the male genitalia [51]. For example, the ventral claspette has a truncate, wide apex, and the basal lobules are moderately expanded laterally with spicules along basal margin; these spicules are moderately long and evenly distributed over the basal surface and radiate in different directions, and the ventral and lateral surfaces of the ventral claspette are covered with short setae. Considering Faran’s 1980 identification key for the female and male genitalia and fourth-instar larva, the key characters used to identify *An. nuneztovari* can be applied as characteristics for the Nuneztovari Complex, since it is likely that the specimens examined by him were a mix of all three species [23]. Also within the Oswaldoi Subgroup we see some support for a grouping of (*An. konderi s.s.*, (*An. konderi* A, *An. konderi* B, *An. oswaldoi* A)) (0.81–1.0 BPP using all three gene regions), discussed below, and for *An. galvaoi* with *An. rangeli* (1.0 BPP using all three gene regions) (Figure 3).

None of the analyses show support for the Strodei Subgroup. The combined DNA analysis supports the remaining members of the Strodei Subgroup if *An. benarrochi* is excluded (1.0 BPP). Most analyses, both DNA and translation, support a grouping of *An. albertoi*, *An. strodei*, *An. arthuri*, and *An. rondoni* (1.0 BPP using all three gene regions), with support from combined DNA analysis for *An. albertoi* with *An. strodei* (1.0 BPP using all three gene regions). There is some, but uneven, support for the Oswaldoi Group (that is the Oswaldoi Subgroup with the Strodei Subgroup) from combined DNA analysis (0.0–1.0 BPP) and from *COI* by itself (0.66–0.78 BPP).

The position of *An. triannulatus* is not resolved with these data. In particular, there is no support for the Oswaldoi Series (including *An. triannulatus*, excluding *An. braziliensis*). In combined DNA analyses *An. triannulatus* goes as sister group to *An. darlingi*, while in translations there is some support for *An. triannulatus* with *An. rangeli* (0.26–0.51 BPP).

#### Argyritarsis section

There is wide and fairly good support for the Albitarsis Group/Complex, from both DNA and translation analysis (1.0 BPP using all three genes, 0.56–0.99 BPP using translations, but 1.0 BPP when using translations with no outgroup; Tables S2 and S3). However, there is no support for the Albitarsis Series, which here would mean the Albitarsis Group with *An. braziliensis*, the latter found to be unstable in our analyses. These and other taxa of the Argyritarsis Section are generally near each other in the tree as a grade, but with no support as a group.

#### Myzorhynchella section

There is good support in all analyses, including analyses of the individual genes, for a crown group composed of all taxa sampled from this section except *An. parvus* (0.72–1.0 BPP using all three gene regions; Table S2). Support for the Myzorhynchella Section, with *An. parvus* basal, is generally good (0.70–1.0 using all three gene regions), but oddly somewhat uneven when the outgroup taxon *An. kompi* is included. None of the three genes separately support the Myzorhynchella Section, but together they do support it (with the *white* gene and the *CAD* gene the placement of *An. parvus* is ambiguous, however the *COI* gene places *An. parvus* in the outgroup).

### Evidence for New Species and a New Subgenus

Identification of the species of the subgenus *Nyssorhynchus* is complicated by the presence of polymorphisms and overlap of morphological characters used in identification keys. Consequently DNA sequence evidence has been used to aid in recognition of species. Marrelli *et al.* (1999) [14] used nucleotide sequences of the second internal transcribed spacer (ITS2) of ribosomal DNA to revise the taxonomic status of *An. oswaldoi*, suggesting that there were at least four sibling species under the name *An. oswaldoi*. Among those species was *An. konderi*, which was subsequently redescribed and removed from the synonymy with *An. oswaldoi* by Flores-Mendoza *et al.* (2004) [52]. Unfortunately Marrelli *et al.* (1999) [14] did not include an *An. konderi* specimen from the type locality (Coari, State of Amazonas, Brazil), and so the molecular identity of *An. konderi* remains undefined. In a recent review, Marrelli *et al.* (2006) [53] showed that one of the sequences generated by Marrelli *et al.* (1999) [14], belonged to an individual of *An. evansae* which had been mistakenly identified as *An. oswaldoi*. More recently, Sallum *et al.* (2008) [36] noted that specimens collected in Acre and preliminarily identified as *An. konderi* could be differentiated by morphological characteristics of the male genitalia from other *An. konderi* specimens and also from that defined by Flores-Mendoza *et al.* (2004) [52]. In addition, differences in the sequences of the ITS2 rDNA corroborated a morphological hypothesis that suggested the presence of undescribed species that could be confused with *An. konderi* when using only female characters. Continuing these studies, Motoki *et al.* (2011) [35] increased *An. konderi* sampling, including representatives of the states of Paraná, Rondônia, Acre and Amapá, and sequences from the ITS2 rDNA, *COI* barcode region of mtDNA and the single copy nuclear *white* gene. As expected, *An. konderi* was confirmed as a species complex. The originality of results obtained by Motoki *et al.* (2011) [35] was that the name *An. konderi* includes at least three species that can be separated by both morphological traits of aedeagus of the male genitalia and by DNA sequence. However, further studies will be necessary to establish accurate comparisons and validate the genetic lineages observed by Sallum *et al.* (2008) [36] and Motoki *et al.* (2011) [35]. Our results in this study described above show support for a grouping of (*An. konderi* A, (*An. konderi s. s.*, *An. konderi* B, *An. oswaldoi* A)) (0.90 BPP; Figure 3 and Figure S1B, see also Table S1 for specimen identity).

The cluster formed by *An. argyritarsis* and *An. argyritarsis s.l.* (MG25_4) seems to be composed of two distinct species; individuals from CE20, CE17 and MG04 represent *An. argyritarsis*, whereas specimen MG25_4 is likely of a distinct species (Figure S1B). Morphological characteristics of the fourth-instar larva, mainly seta 3-T of the metathorax and seta 1-I of the abdominal segment I, allow separation of *An. argyritarsis s.l.* MG25_4 from the remaining *An. argyritarsis* (CE20, CE17 and MG04). Additionally, female abdominal scales key out the individual as *An. sawyeri*, whereas larval setal characteristics do not key out the specimen either as *An. argyritarsis* or *An. sawyeri*. Considering morphological differences observed in the specimen MG25_4 regarding to both *An. argyritarsis* and *An. sawyeri*, and to avoid premature species definition, that individual was assigned to the morphologically closest taxon, until male and female linked to larval and pupal exuviae are obtained in field collections, and DNA sequence can be associated to specimen identified with accuracy.

Until recently the Myzorhynchella Section consisted of four named species; currently it is composed of six valid taxa – *An. antunesi*, *An. guarani*, *An. lutzii*, *An nigritarsis*, *An. pristinus* and *An. parvus*. *Anopheles guarani* was resurrected from synonymy of *An. lutzii* by Nagaki *et al.* (2011) [40], and *An. pristinus* was described by Nagaki and Sallum (in Nagaki *et al.* 2010, [41]). Recently, Bourke *et al.* (2011) [54] suggested that individuals identified as *An. lutzii*A325 and *An. lutzii*B369 may represent two unnamed species. Additional specimens obtained in the same geographical region of *An. lutzii*A325 and *An. lutzii*B369 confirmed that they are two unnamed taxa that can be misidentified as *An. lutzii* if the identification is based only on external characters of the female. One cluster of individuals (including RS19 and RS33, tentatively labelled as *An. lutzii* A in Figure 3 ) seems to be more closely related to *An. lutzii s.s.* than to any other species of the Myzorhynchella Section. The second cluster, including RS16a and RS16b, together with B369, tentatively labelled *An. lutzii* B in Figure 3, is sister to *An. antunesi* (see Table S1 for specimen identity). The COI barcode distances between B369 and the two RS16 specimens is 4.9% (Table S5), suggesting that B369 is a species separate from the other two. The hypothesis of three unnamed taxa is corroborated by morphological traits of the male genitalia and fourth-instar larva. The new species will be formally described and named in a further study by one of the authors.

Other specimens with large intraspecific distances (Table S5) which would be candidates for being considered separate species include An_parvus_MG07_9_1, An_triannulatus_ES03_03_01, An_oswaldoi_SP22_9, and An_evansae_SP12_44.

If we look at pairs with the largest (>16%) K2P distances we can see that one member of the pair was always a specimen of *An. parvus*. These distances are larger than seen in intra-subgenus distances where one member of the pair was from the outgroup taxa *An. cruzii* or *An. intermedius*; these large genetic distances suggest that *An. parvus* should be considered a distinct subgenus. This is corroborated by morphological differences. Differences in the eggs as seen with SEM [55] and in male genitalia traits can easily separate *An. parvus* from all other species on the subgenus *Nyssorhynchus*. For example, the presence of spicules in the mesal margin of the ventral lobule of the ventral claspette is a characteristic that is not observed in any species of the Myzorhynchella section, and the parabasal lobule is curved, not straight as in species of the *Nyssorhynchus*. *An. parvus* was described as a species of the genus *Myzorhynchella* (currently a section), which has *Anopheles niger* as the type species. The latter species is in synonymy with *An. lutzii*, and consequently the name Myzorhynchella cannot be used for *An. parvus* if it is determined to be a species of a distinct subgenus. Whether Myzorhynchella is a section of the subgenus *Nyssorhynchus* or a subgenus of the genus *Anopheles* is an open question to be answered in further studies.

Based on phylogenetic analysis (Figure S1), it was speculated that *An. nuneztovari* specimen RO2_13 was misidentified, and indeed this specimen seems to be a representative of *An. goeldii* that was misidentified by morphological traits as *An. nuneztovari*. Consequently, both species may be sympatric in Rondônia state, Brazil, extending geographical distribution of *An. goeldii* to the western Amazon River basin.

### Conclusions

1. **A single gene region is often not sufficient to place specimens in morphologically defined species.** The *COI* barcode region is for species identification, and has been used to not only corroborate morphologically defined species but also to define new species in the *Anopheles albitarsis* Complex [37]. We have tested the ability of *COI*, *white*, and *CAD* to group specimens known to be in the same species together, and have shown that individual genes often are unable to do this, while the concatenated genes are much better at delineating species.
  The method of analysis affects the veracity of the observation above. Using the simple K2P/NJ method there was only a small improvement with an increase in the amount of sequence data. However, with a Bayesian analysis and better fitting models there was a greater improvement (Table 2).

1. **We need more sequence data in order to show higher level groupings.** It may be that the noise and uncertainty that we see is due to systematic error, in which case we need better methods or models, or to poor sampling, in which case we need more sequence data. If few data give us poor resolution or poor support, but having more sequence data allows us to obtain results that are consistent with well-established relationships, that argues that we need primarily more sequence data.
  We have examples from this study that support this. For example, we have some support for Oswaldoi Subgroup from the combined DNA analysis, but not from any of the three genes individually. Another example is that none of the three genes separately support the Myzorhynchella Section, but together they do support it. However, our study leaves too many unanswered questions regarding the phylogenetic relationships in the subgenus *Nyssorhynchus*, even with three genes. It is an easy prediction that more sequence information will help in this regard, and an obvious target would be to get mitochondrial genomes, which have helped resolve similar problems elsewhere [56]–[59].
  Translations can be useful for deeper phylogenetic distinctions. Analysis of the translations, that is analysis at the amino acid level, can give results that make more sense than the DNA analysis; for example the position of *An. kompi* in the outgroup was seen with translations but not with DNA. However, the translation sequences are more similar to each other, as is expected within a subgenus, and so there is poor resolution between closely related taxa. What we see in this case using translations is an unresolved comb phylogeny.

1. We propose that *An. dunhami* should be part of the Nuneztovari Complex.
2. We have some phylogenetic evidence for new species, corroborated by morphological evidence.
  - *An. argyritarsis s.l.* may represent a new species, morphologically similar to both *An. argyritarsis* and *An. sawyeri*.
  - Specimens of the tentatively named *An. oswaldoi* A from Pará state and Acre (AC18) are likely representatives of an undescribed species that is morphologically similar to *An. oswaldoi*.
  - *An. konderi* from Amapa state and AC18_16 from Acre state seem to belong to two species closely related to *An. konderi* that have not yet been formally named. Specimens of *An. konderi* from Rondônia and Paraná state may represent *An. konderi s.s.* because the type locality is Coari, Amazonas state, situated south of the Amazon River basin. Additionally, morphological comparison of male genitalia of one specimen from Coari collected by Flores-Mendoza in 1998 shows that representatives from Rondônia and Paraná states are morphologically more similar to that of Coari than to those from individuals from Amapa and Acre states.
  - Tentatively-named *An luzii* A, B, and B369 appear to be separate species.
3. There are several specimens with large intraspecific K2P distances (>3%) that are candidates for being considered separate species. These include An_parvus_MG07_9_1, An_triannulatus_ES03_03_01, An_oswaldoi_SP22_9, and An_evansae_SP12_44.
4. Based on morphological differences and very large (>16%) K2P distances, *An. parvus* should possibly be considered a separate subgenus of *Anopheles*.

## Materials and Methods

### Ethics Statement

All necessary permits were obtained for the described field studies. Collections were made under permanent permit number 16938-1 from Instituto Brasileiro do Meio Ambiente e dos Recursos Naturais Renováveis (IBAMA) to Maria Anice M. Sallum. Specific permission was not required for these locations as permission to collect was granted under the permanent permit. The collection locations were not privately owned or protected in any way. The field studies did not involve protected or endangered species.

### Taxon Sampling

The species sampled for this study and the sources of specimens are listed in Table S6. Larvae and pupae were either collected from field habitats or obtained from link-reared offspring of blood fed females collected in the field. Both larvae and pupae were maintained in the laboratory to obtain adult males and females associated with larval and pupal exuviae. Freshly emerged mosquitoes were quickly anesthetized with ethyl acetate vapors, and either kept separate in minute plastic vials in silica gel or individually frozen at −80°C. Species identification was based on either adult male genitalia or fourth-instar larval characteristics. For few taxa, identification was also based on the external morphology of the eggs observed in a Jeol JSM-6460 scanning electron microscope (SEM) (Jeol Ltd., Akishima, Japan) as reported by Sallum *et al.* (2010) [33] and Nagaki *et al.* (2010) [41].

DNA extraction. DNA was extracted from whole mosquitos, following the insect DNA extraction protocol provided by the QIAgen DNeasy Blood and Tissue Kit (QIAgen Ltd., Crawley, UK).

### DNA Amplification and Sequencing

#### *COI* mtDNA

The primers LCO1490 (5′ GGT CAA CAA ATC ATA AAG ATA TTG G 3′) and HCO2198 (5′ TAA ACT TCA GGG TGA CCA AAA AAT CA 3′) (Table S7) were used to amplify about 650 base pairs of the mitochondrial cytochrome subunit I (COI mtDNA). Each PCR reaction contained 1 *µ*l template DNA (about 1/200th to 1/1000th of the amount extracted from a single specimen); 20 mM Tris-HCl, pH 8.4; 50 mM KCl; 1.5 mM MgCl2; 5 picomoles of each primer; 200 *µ*M each dNTPs; and 2.5 U Taq polymerase; and the remaining volume of ultra pure water up to 25 *µ*l. PCR amplification protocol consisted of a 3-min denaturation at 94°C and 35 cycles at 94°C, 55° and 72°C for 1 minute each, followed by a 7 minute extension at 72°C.

#### Nuclear *white* gene

Amplification of about 700 base pairs of the nuclear single copy *white* gene was obtained using the primers WZ2E and WZ11X (Table S7). Each PCR reaction contained 1–2 *µ*l template DNA (about 1/100th to 1/200th of the amount extracted from a single specimen); 20 mM Tris-HCl, pH 8.4; 50 mM KCl; 1.5 mM MgCl2; 2.5 µl DMSO; 100 picomoles of each primer; 200 *µ*M each dNTPs; 2.5 U Taq polymerase; and the remaining volume of ultra pure water up to 25 *µ*l. PCR amplification protocol consisted of a 3-minute denaturation at 94°C, 35 cycles at 94°C for 30 sec, 50°C for 1 min and 72°C for 2 min each, followed by a 7-min extension at 72°C. PCR amplicons obtained from six females were purified using PEG precipitation (20% polyethylene glycol 8000/2.5 M NaCl) and cloned into pGem-T Easy Vector (Promega, Madison, WI, USA). Two to four positive clones were sequenced.

#### Nuclear *CAD* gene

Amplification of about 648–895 base pairs of the nuclear single copy *CAD* gene was obtained using the primers listed in Table S7. Each PCR reaction contained 2 to 4 *µ*l template DNA (about 1/50th to 1/100th of the amount extracted from a single specimen); 20 mM Tris-HCl, pH 8.4; 50 mM KCl; 1.5 to 2.4 mM MgCl2; 2.5 *µ*l DMSO; 100 picomoles of each primer; 200 µM each dNTPs; 2.5 U Taq polymerase; and the remaining volume of ultra pure water up to 25 to 50 *µ*l. PCR amplification protocol consisted of a 2 to 4-min denaturation at 94°C, 35 cycles at 94°C for 20 to 30 sec, 50°C to 57°C for 30 sec and 72°C for 1 min each, followed by a 7-min extension at 72°C. For the PCR employing CAD338F_M13 and CAD680R_M13, the protocol consisted of a 3-min denaturation at 94°C, 35 cycles at 94°C for 1 min, 56°C for 1 min and 72°C for 1 min each, followed by a 7-min extension at 72°C. PCR amplicons obtained from 13 individuals were purified using PEG precipitation (20% polyethylene glycol 8000/2.5 M NaCl) and cloned into pGem-T Easy Vector (Promega, Madison, WI, USA). One to four positive clones were sequenced.

PCR products of *white*, *CAD*, and *COI* genes were electrophoresed in 1% TAE agarose gels stained with GelRedTM Nucleic Acid Gel Stain (Biotium Inc., Hayward, USA). All sequencing reactions were carried out in both directions using ABI Big Dye Terminator Kit v.3.1 (PE Applied Biosystems, Warrington, England). For *COI* we employed the same primers used for PCR, whereas for the *white* and *CAD* genes, primers were variable and are listed in Table S7. Sequencing reaction consisted of 0.5 *µ*l of Big Dye Terminator Ready Reaction Mix; 3 *µ*l of 5X sequence dilution-buffer [5 mM MgCl2, 200 mM Tris-HCl, pH 9.0]; 3.6 picomoles of R or F primer; 10 ng of PEG purified PCR product, ultra pure water up to 10 *µ*l. Sequencing reactions were purified in Sephadex G50 columns (GE Healthcare). Sequences were analyzed on an ABI Prism 3130 – Avant Genetic Analyzer (Applied Biosystems, Foster City, CA, U.S.A.), and edited using Sequencher for Windows version 4.9 (Gene Codes Corporation, Ann Arbor, USA).

#### DNA vouchers

Template DNA from this study is retained at −70°C in the Faculdade de Saúde Pública (FSP-USP), São Paulo, Brazil, for future reference (DNA reference numbers are in Table S6). Immature and male genitalia slide of the specimens used for DNA extraction are deposited in the FSP-USP collection.

### Sequence Data Preparation and Analysis

Among the 144 specimens, all had a *COI* sequence, 137 had a *white* sequence, and 129 had a *CAD* sequence (Table S8). DNA sequences were translated using Genewise v 2.2.0 [60], [61], using closely related protein guide sequences from GenBank and appropriate genetic codes. The resulting amino acid sequences were aligned with Clustalo (v 1.0.2, [62]). Inspection of the alignments showed that the *white* gene and the *CAD* gene had unreliable alignment sites, which were masked out by hand. The codons used to make the translations were back-aligned to the protein alignments, and correspondingly masked. There was an intron in the *white* gene (although not all sequences had that intron); separate alignment of the intron DNA sequence with Muscle [63] did not give a satisfactory alignment, so the intron was not used further.

#### Analysis using K2P/NJ

Pairwise Kimura 2-parameter (K2P) [64] pairwise distances were calculated from the aligned COI barcode region and the three aligned concatenated genes, both without *An. kompi*, using PAUP* [65]. Trees were made from these distances with the neighbour joining (NJ) algorithm [66], again as implemented in PAUP*.

#### Bayesian phylogenetic analysis

The aligned concatenated genes are characterized in Table 1. Notice that there are no informative sites in the *COI* second codon position; it was not used in the Bayesian analyses.

Preliminary analysis showed that partitioning by gene and by codon was better than partitioning by either alone. Models were chosen for the eight partitions in isolation, aided by Modeltest v 3.7 [67]. Modeltest was done on the separate partitions after removing blank sequences, homogenizing duplicate sequences, and then using only likelihood informative sites. The models suggested are tabulated in Table S9, together with the AIC weight. Often the suggested model was not implemented in the software used to do the analysis (MrBayes or p4), and so the model actually used is also shown, together with its AIC weight. Often the AIC weights were low (generally 0.2 or less), indicating ambiguity in model choice.

Only the 81 topology informative sites of the 766 sites in the concatenated translation were used. In these sequences 15 of the 144 sequences had more than 40 gaps in the alignment of 81 sites, and those gappy sequences were removed as being too short. However, one of those short sequences was *An. kompi*, one of the outgroup sequences, and this sequence was retained for some analyses even though it was short. Finally, duplicate sequences were homogenized, leaving 106 sequences (including *An. kompi*). Model choice was aided by Prottest v3.0 [68], which indicated that the JTT+G model was best by the AIC criterion, with an AIC Weight of 0.999.

## Supporting Information

Figure S1 **Phylogenetic trees from DNA.** Consensus trees of Bayesian and K2P/NJ analysis of individual and concatenated gene regions. See the caption in the Figure for details of analysis. (A) Analysis of DNA of all three gene regions, including *An. kompi* in the the outgroup. (B) Analysis of all three gene regions, not including *An. kompi* in the the outgroup. (C) Analysis of the *white* gene only. (D) Analysis of the *CAD* gene only. (E) Analysis of the *COI* barcode region only. (F) K2P/NJ tree of the COI barcode region, not including *An. kompi* in the outgroup. (G) K2P/NJ tree of the DNA from all three sequences, not including *An. kompi* in the outgroup.
(PDF)

Figure S2 **Phylogenetic trees from protein translations.** Consensus trees of Bayesian analysis of translations of concatenated gene regions. See the caption in the Figure for details of the analysis. (A) Analysis of protein translations of all three gene regions, including *An. kompi* in the the outgroup. (B) Analysis of protein translations of all three gene regions, not including *An. kompi* in the the outgroup. (C) Analysis of protein translations of all three gene regions, not including the outgroup.
(PDF)

Table S1 Species and specimens. A table showing the specimens used in this study, and the morphologically-based species identification.
(PDF)

Table S2 Support for higher-level groupings from Bayesian analyses of DNA sequences. Support for species is shown in Table S6. Support is shown as Bayesian posterior probability as taken from the posterior distribution; that is from the sampled trees, not from a consensus tree.
(PDF)

Table S3 Support for species and higher-order groupings from gene translations. Support is shown as Bayesian posterior probability as taken from the posterior distribution; that is from the sampled trees, not from a consensus tree.
(PDF)

Table S4 Support for species from three concatenated gene regions and from single gene regions only. Support is shown as Bayesian posterior probability as taken from the posterior distribution; that is from the sampled trees, not from a consensus tree. Support for higher-level groupings is shown in Table S4.
(PDF)

Table S5 Intraspecifc K2P distances greater than 3%: Possible candidates for new species. One of the pair would be a possible candidate for placement in another species.
(PDF)

Table S6 Specimen collection locations and details.
(XLS)

Table S7 DNA amplification primers.
(DOC)

Table S8 Specimens and sequences used. A table showing which sequences were used in the analysis, and what name was given to those sequences.
(PDF)

Table S9 Model choice. Tables showing the models suggested by Modeltest and the models that were used in the model-based DNA analyses. The Akaike weights were often low, showing ambiguity in the model choice.
(PDF)
