# Supplementary material for: Phylogenetic Analysis and DNA-based Species Confirmation in Anopheles (Nyssorhynchus)
Source: PLoS One. 2013 Feb 4;8(2):e54063. doi: 10.1371/journal.pone.0054063 (PMC3563636; doi:10.1371/journal.pone.0054063)

ALBIMANUS SECTION
- OSWALDOI SERIES
  - Oswaldoi Group
    - Oswaldoi Subgroup
      - *Anopheles oswaldoi ss*
      - *Anopheles oswaldoi* A
      - *Anopheles galvaoi*
      - *Anopheles evansae*
      - *Anopheles konderi* A
      - *Anopheles konderi ss*
      - *Anopheles konderi* B
      - *Anopheles rangeli*
      - *Anopheles dunhami*
      - Nuneztovari Complex
        - *Anopheles nunzetovari*
        - *Anopheles goeldii*
    - Strodei Subgroup
      - *Anopheles albertoi*
      - *Anopheles arthuri*
      - *Anopheles strodei*
      - *Anopheles rondoni*
      - *Anopheles benarrochi*
      - *Anopheles* CP Form
  - Triannulatus Group
    - *Anopheles triannulatus*

ARGYRITARSIS SECTION
- ARGYRITARSIS SERIES
  - Argyritarsis Group
    - *Anopheles argyritarsis*
  - Lanei Group
    - *Anopheles lanei*
  - Pictipennis Group
    - *Anopheles atacamensis*
  - Darlingi Group
    - *Anopheles darlingi*
- ALBITARSIS SERIES
  - Albitarsis Group/Complex
    - *Anopheles albitarsis ss*
    - *Anopheles oryzalimnetes*
    - *Anopheles deaneorum*
    - *Anopheles marajoara*
  - Braziliensis Group
    - *Anopheles braziliensis*

MYZORHYNCHELLA SECTION
- *Anopheles antunesi*
- *Anopheles guarani*
- *Anopheles lutzii ss*
- *Anopheles lutzii* B
- *Anopheles lutzii* A
- *Anopheles parvus*
- *Anopheles pristinus*

OUTGROUP
- *Anopheles cruzii*
- *Anopheles intermedius*
- *Anopheles kompi*

Figure S1A–E. Bayesian analyses using both MrBayes and p4 were run for 10 million generations, with each codon position of each gene given its own model characteristics in a multi-partition model as described in Supplemental Tables S2A and S2B (although the COI second codon position 2 had no informative sites and so was not used). MrBayes used an MCMCMC with 4 chains; p4 used a single chain (*ie* no MCMCMC). MrBayes runs collected 1001 samples, and used a burnin of 751 samples. The p4 analysis collected 2000 samples and used a burnin of 1500 samples. Two runs were made using MrBayes, and 5 runs with p4; separate results can be seen in Supplemental Tables S4 and S6. The figures here (S1A–E) are consensus trees made from pooled samples. Internal nodes are decorated with posterior probabilities, expressed as percent.
Figure S1F, S1G. K2P/NJ trees. Pairwise K2P distances were used to to make a neighbor-joining tree, as shown. Negative branch lengths were collapsed to zero length.

Supplementary Figure S1A. Analysis of DNA of all three genes, including An. kompi in the the outgroup.

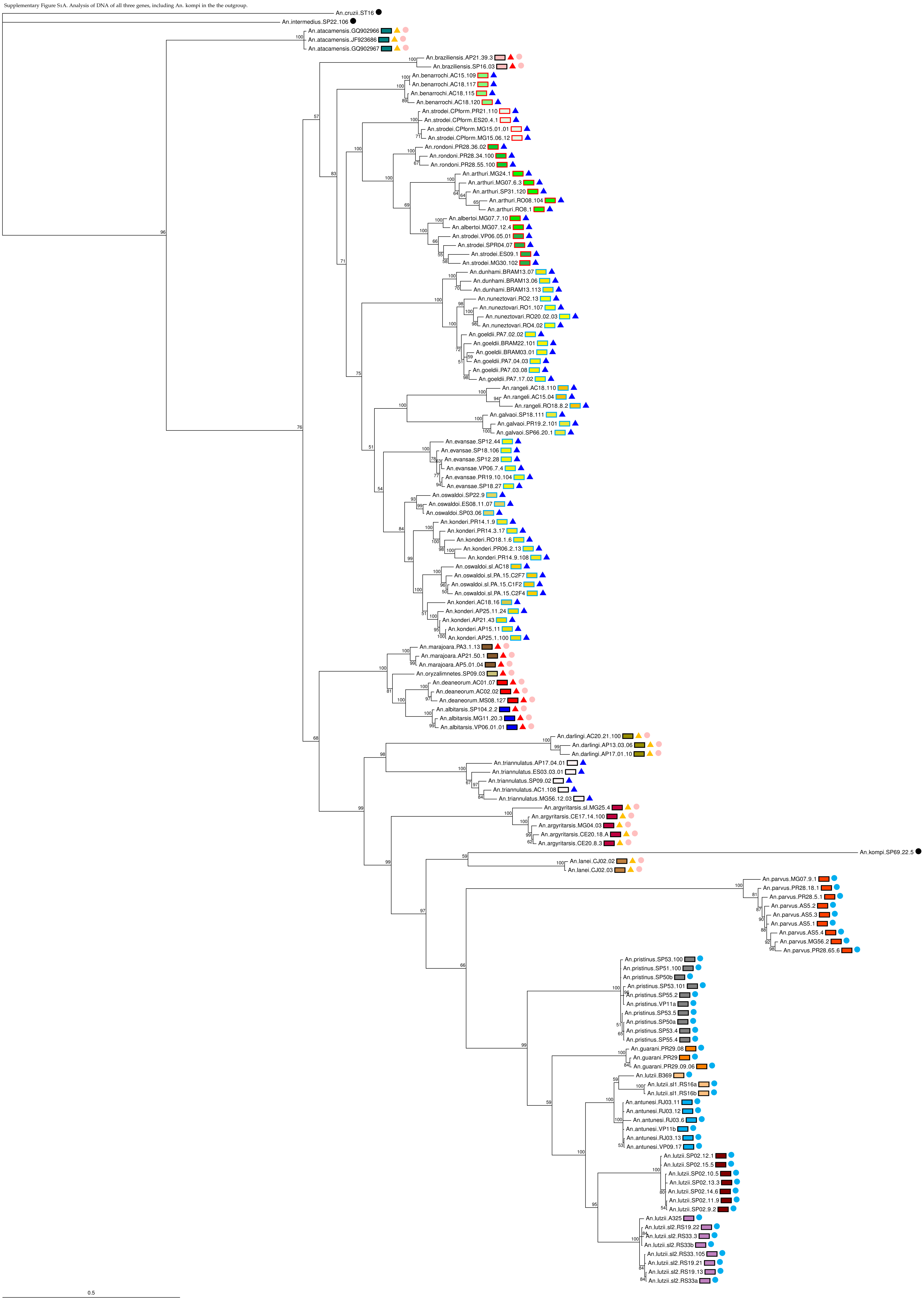

Supplementary Figure S1B. Analysis of DNA of all three genes, not including An. kompi in the the outgroup.

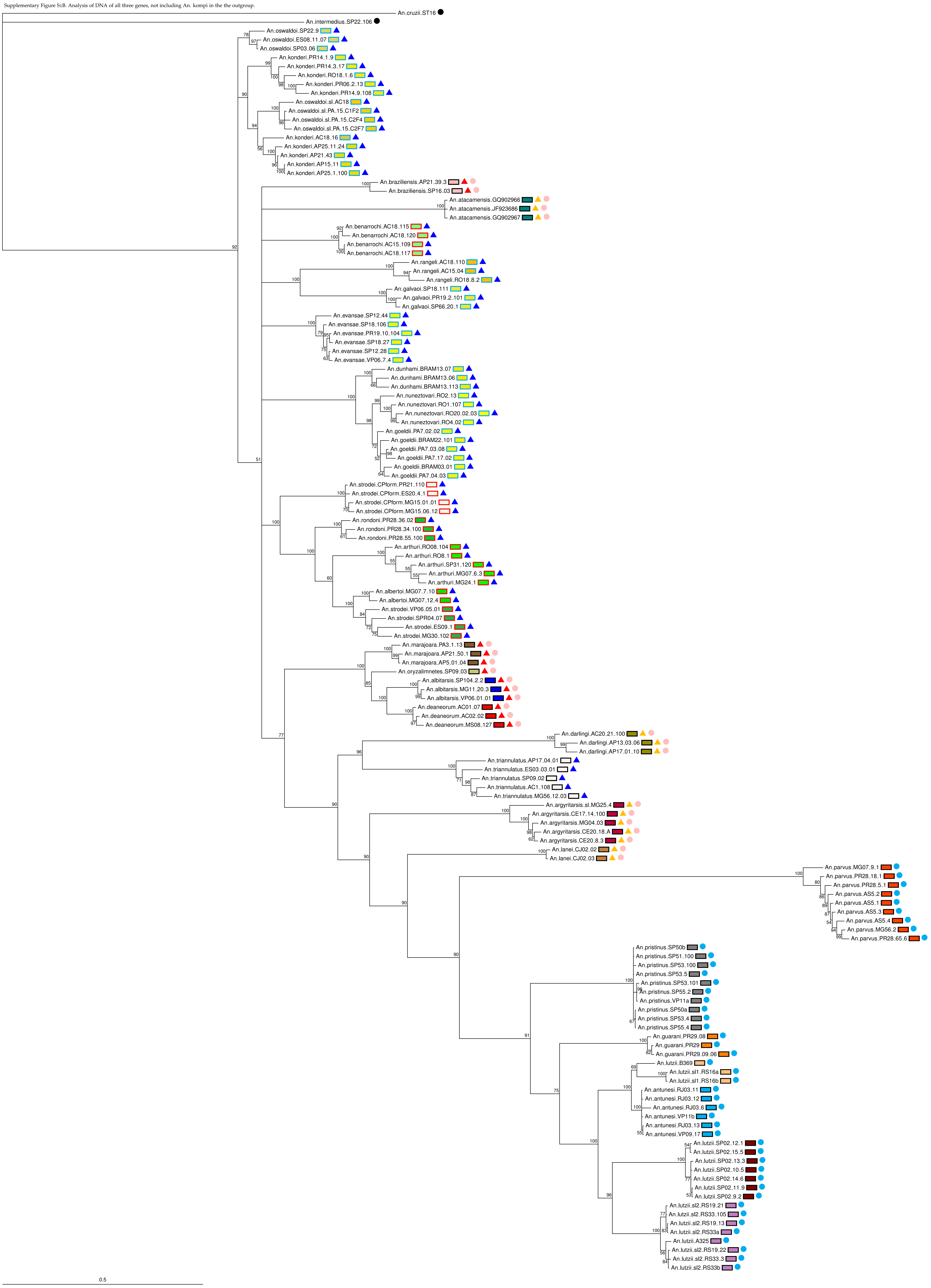

Supplementary Figure S1C. Analysis of DNA of the *white* gene only.

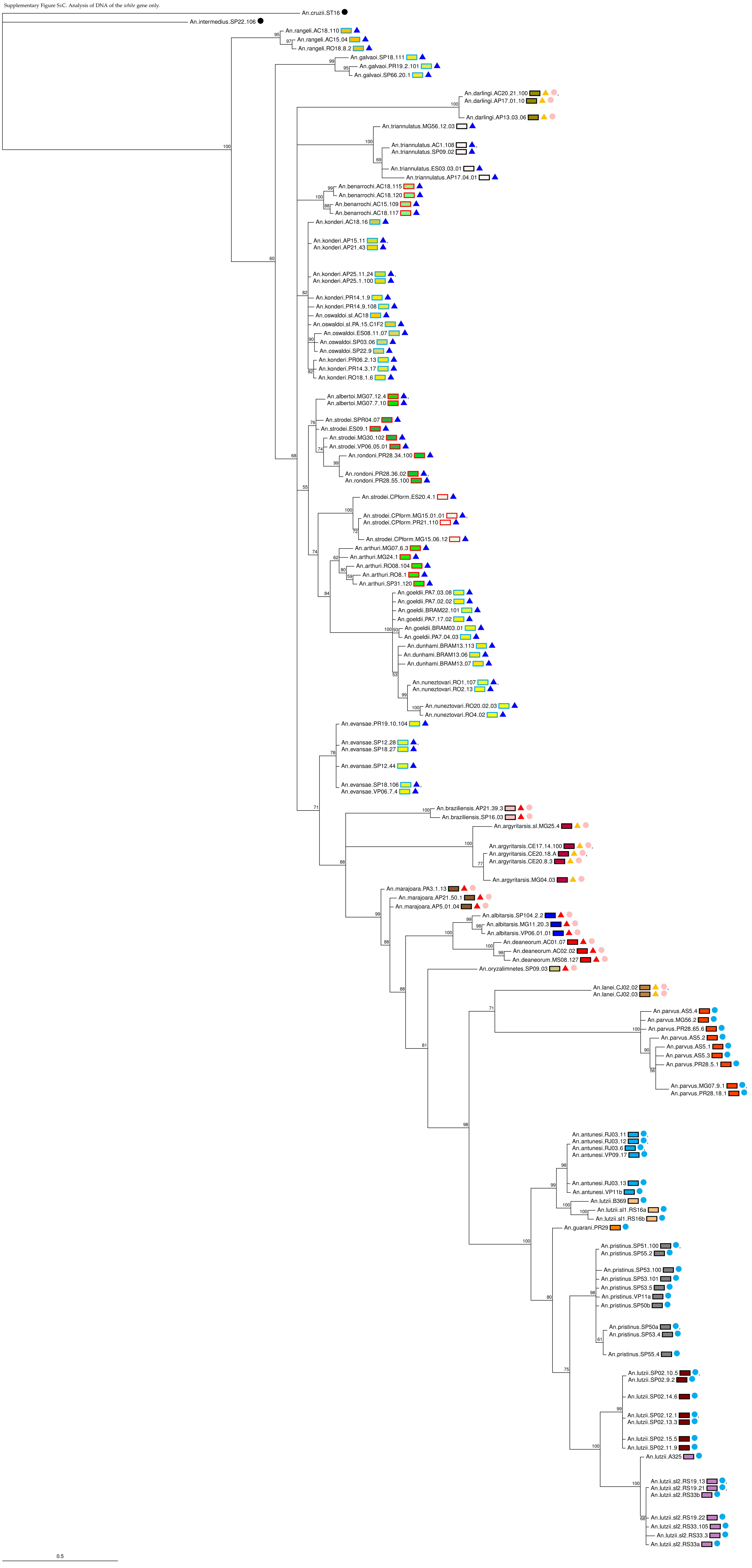

Supplementary Figure S1D. Analysis of DNA of the *CAD* gene only.

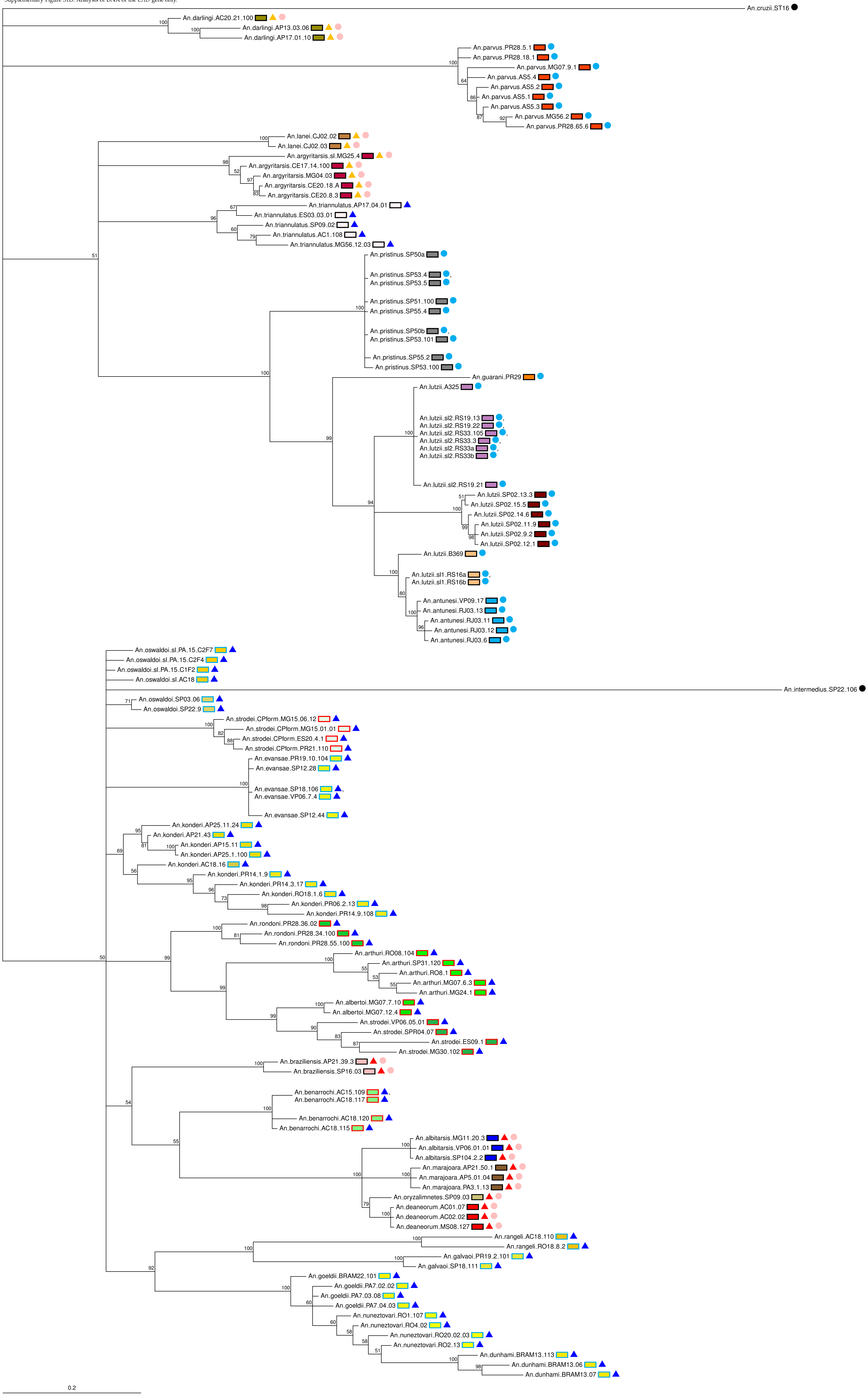

Supplementary Figure S1E. Analysis of DNA of the *COI* gene only.

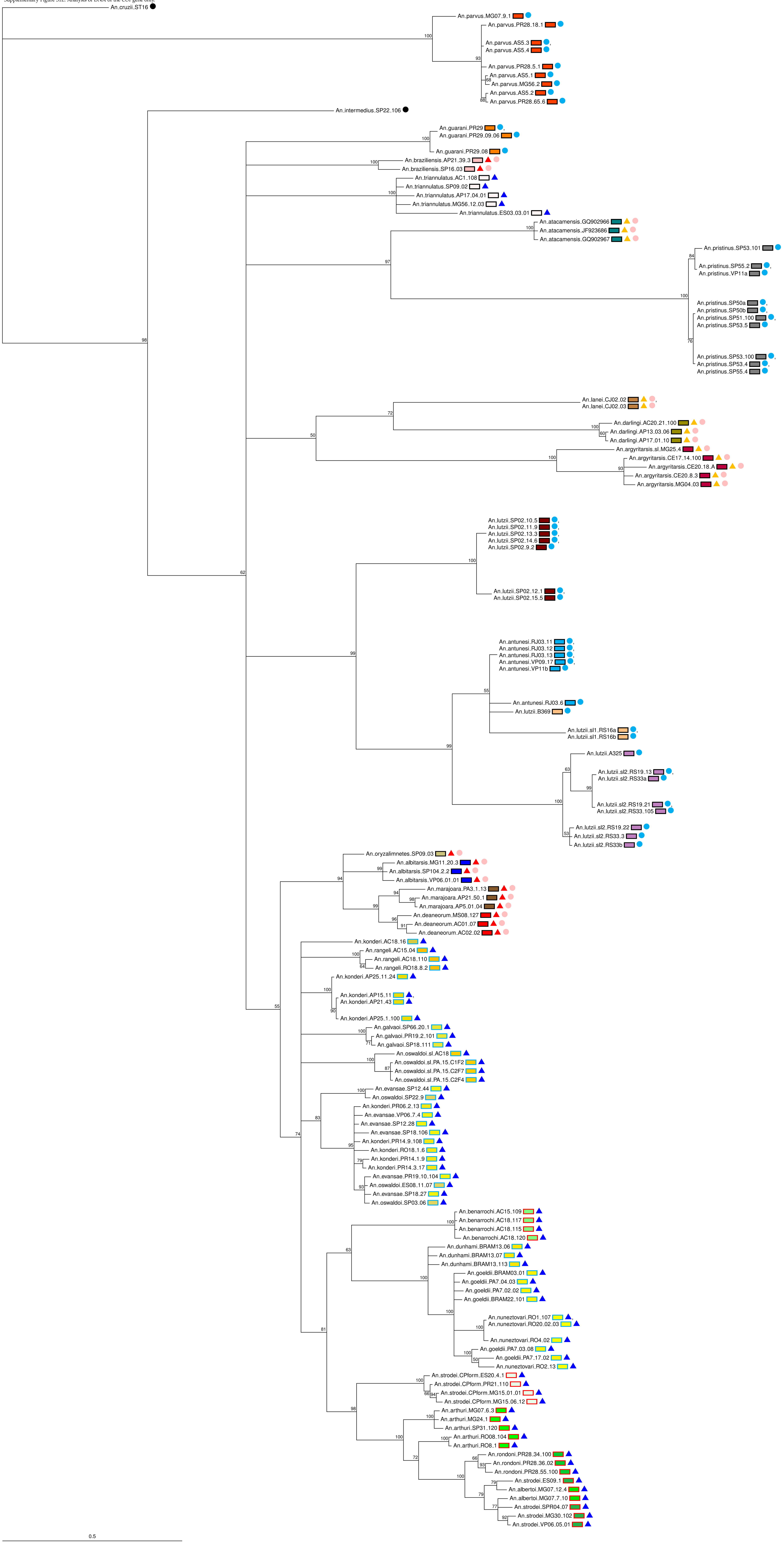

Supplementary Figure S1F. K2P/NJ tree of the COI barcode region, not including *An. kompi* in the outgroup.

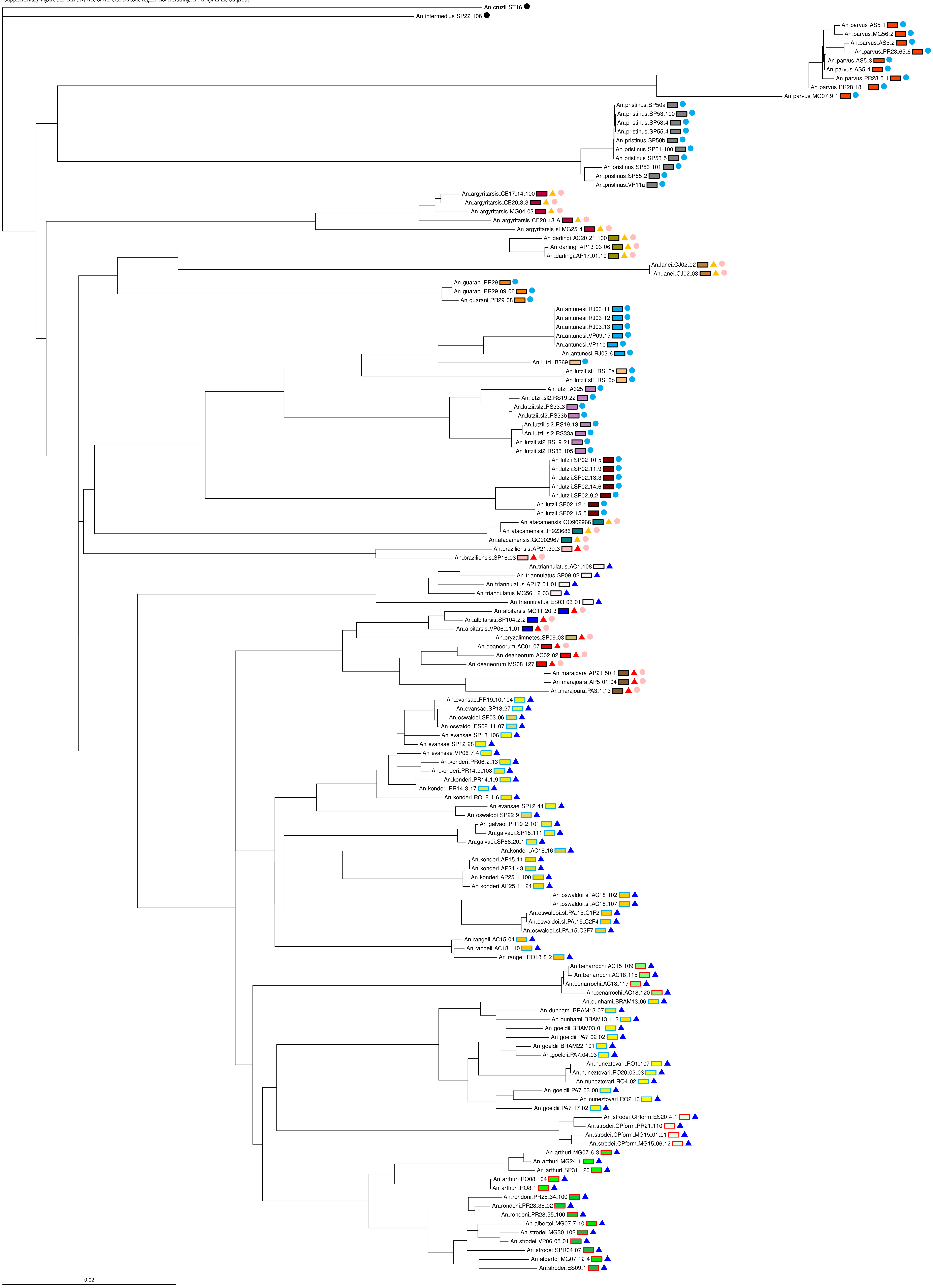

Supplementary Figure S1G. K2P/NJ tree of the DNA from all three sequences, not including *An. kompi* in the outgroup.

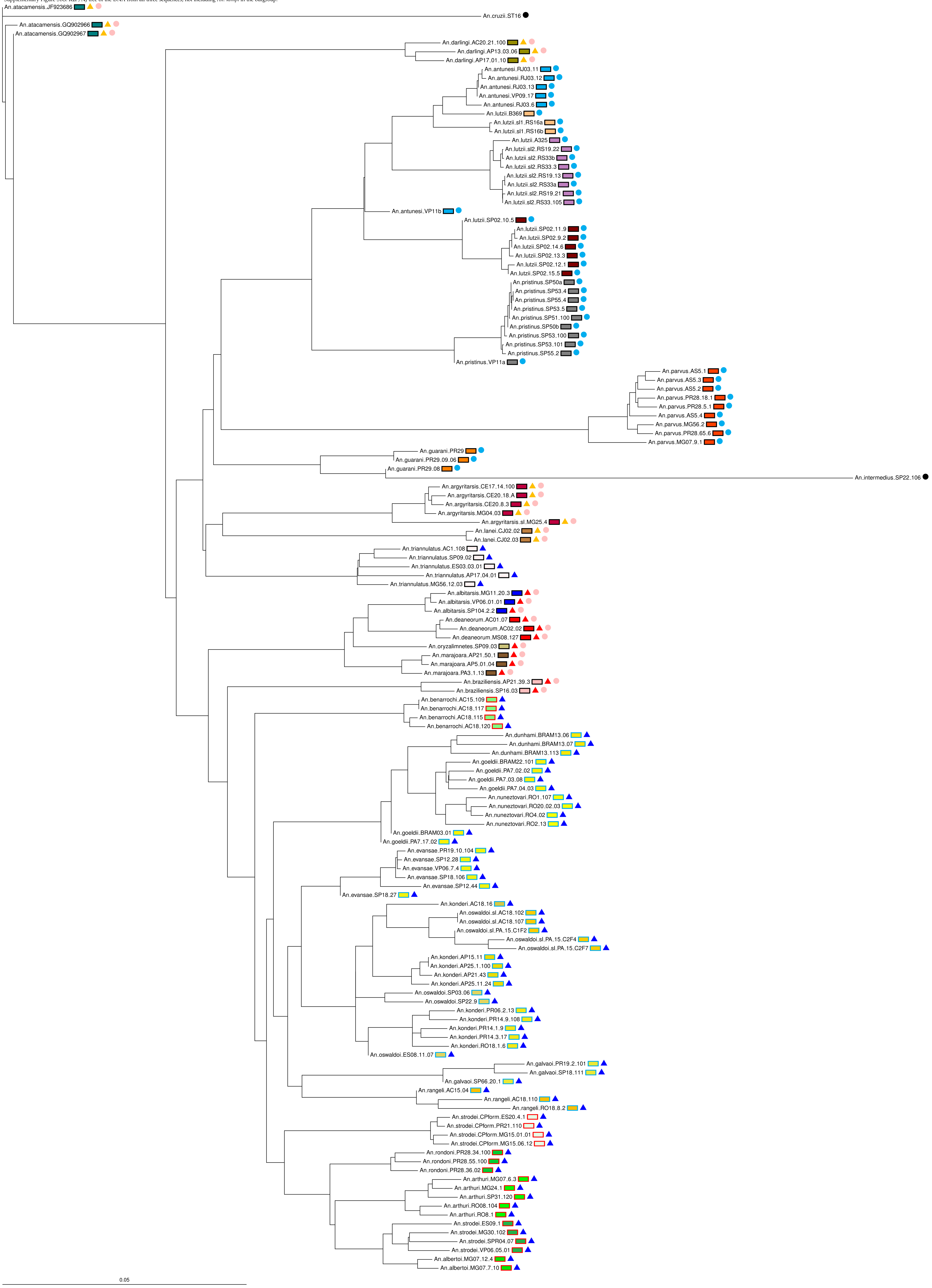

Supplement: Figure S1 — Phylogenetic trees from DNA. Consensus trees of Bayesian and K2P/NJ analysis of individual and concatenated gene regions. See the caption in the Figure for details of analysis. (A) Analysis of DNA of all three gene regions, including An. kompi in the the outgroup. (B) Analysis of all three gene regions, not including An. kompi in the the outgroup. (C) Analysis of the white gene only. (D) Analysis of the CAD gene only. (E) Analysis of the COI barcode region only. (F) K2P/NJ tree of the COI barcode region, not including An. kompi in the outgroup. (G) K2P/NJ tree of the DNA from all three sequences, not including An. kompi in the outgroup. (PDF) [file pone.0054063.s001.pdf]
