# Supplementary material for: Phylogenetic Analysis and DNA-based Species Confirmation in Anopheles (Nyssorhynchus)
Source: PLoS One. 2013 Feb 4;8(2):e54063. doi: 10.1371/journal.pone.0054063 (PMC3563636; doi:10.1371/journal.pone.0054063)

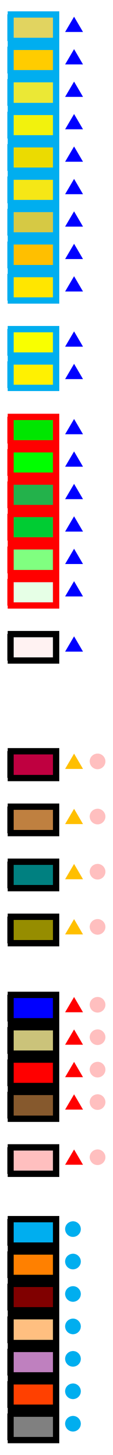

ALBIMANUS SECTION
- OSWALDOI SERIES
  - Oswaldoi Group
    - Oswaldoi Subgroup
      - *Anopheles oswaldoi ss*
      - *Anopheles oswaldoi* A
      - *Anopheles galvaoi*
      - *Anopheles evansae*
      - *Anopheles konderi* A
      - *Anopheles konderi ss*
      - *Anopheles konderi* B
      - *Anopheles rangeli*
      - *Anopheles dunhami*
      - Nuneztovari Complex
        - *Anopheles nuneztovari*
        - *Anopheles goeldii*
    - Strodei Subgroup
      - *Anopheles albertoi*
      - *Anopheles arthuri*
      - *Anopheles strodei*
      - *Anopheles rondoni*
      - *Anopheles benarrochi*
      - *Anopheles* CP Form
  - Triannulatus Group
    - *Anopheles triannulatus*

ARGYRITARSIS SECTION
- ARGYRITARSIS SERIES
  - Argyritarsis Group
    - *Anopheles argyritarsis*
  - Lanei Group
    - *Anopheles lanei*
  - Pictipennis Group
    - *Anopheles atacamensis*
  - Darlingi Group
    - *Anopheles darlingi*
- ALBITARSIS SERIES
  - Albitarsis Group/Complex
    - *Anopheles albitarsis ss*
    - *Anopheles oryzalimnetes*
    - *Anopheles deaneorum*
    - *Anopheles marajoara*
  - Braziliensis Group
    - *Anopheles braziliensis*

MYZORHYNCHELLA SECTION
- *Anopheles antunesi*
- *Anopheles guarani*
- *Anopheles lutzii ss*
- *Anopheles lutzii* B
- *Anopheles lutzii* A
- *Anopheles parvus*
- *Anopheles pristinus*

OUTGROUP
- *Anopheles cruzii*
- *Anopheles intermedius*
- *Anopheles kompi*

Figure S2A–C. Bayesian phylogenetic analysis of protein translations of the three gene regions. MCMC analysis used both MrBayes with the JTT+G model, and p4 with the JTT+F+G model ("+F implying using emprical frequencies; this model aspect is not available in MrBayes). MrBayes used an MCMCMC with 4 chains, and p4 used a single chain (*ie* no MCMCMC). The consensus tree of the pooled post burnin samples is shown, with internal nodes decorated with posterior probability expressed as percent. Separate results can be seen in Table S5. (A) Analysis of protein translations of all three gene regions, including *An. kompi* in the the outgroup. This used two runs using MrBayes, and 10 runs using p4, both for 10 million generations. The MrBayes runs took 2001 samples and used a burnin of 1501 samples, and the p4 runs took 2000 samples and used a burnin of 1500 samples. (B) Analysis of protein translations of all three gene regions, not including *An. kompi* in the the outgroup. This used two runs for MrBayes, and 5 runs for p4, both for 5 million generations. The MrBayes runs took 1001 samples with a burnin of 751 samples. The p4 runs took 5000 samples and used a burnin of 4000 samples. The polytomy prior was used in p4. (C) Analysis of protein translations of all three gene regions, not including the outgroup. Conditions were the same as for (B) except that 2001 samples were taken in the MrBayes runs, with a burnin of 1501 samples.

Supplementary Figure S2A. Analysis of protein translations of all three gene regions, including *An. kompi* in the the outgroup.

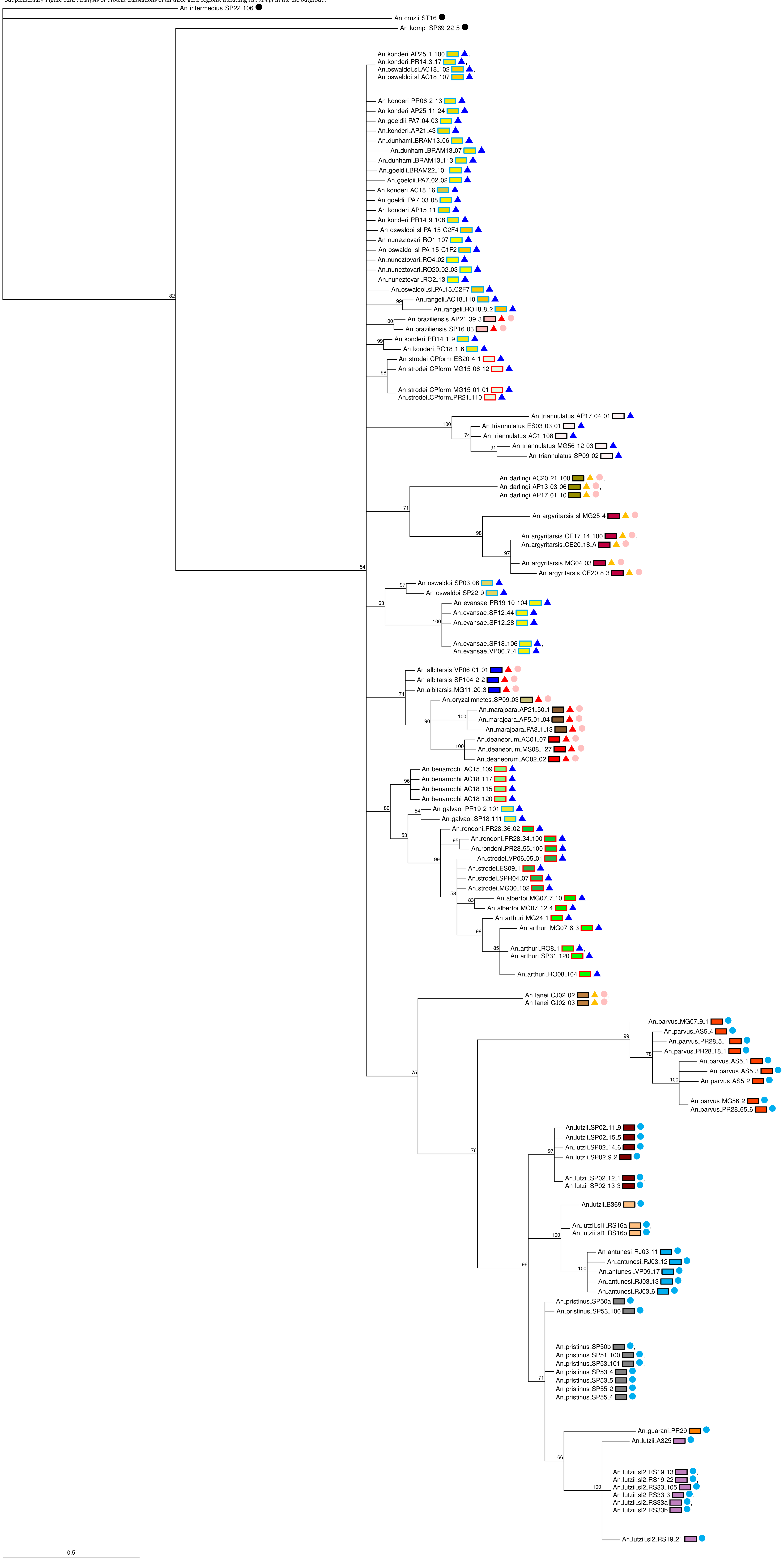

Supplementary Figure S2B. Analysis of protein translations of all three gene regions, not including *An. kompi* in the the outgroup.

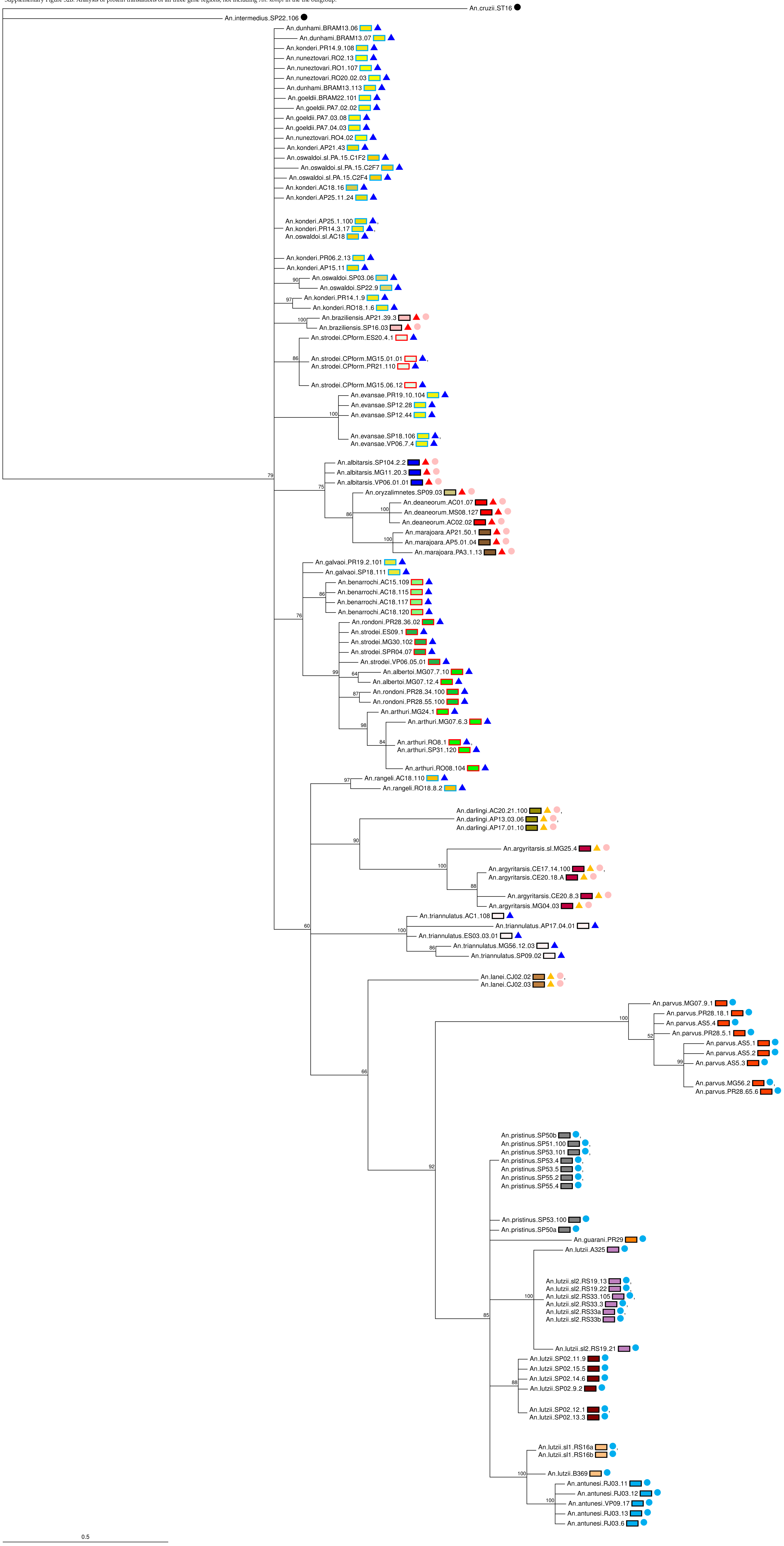

Supplementary Figure S2C. Analysis of protein translations of all three gene regions, not including the outgroup.

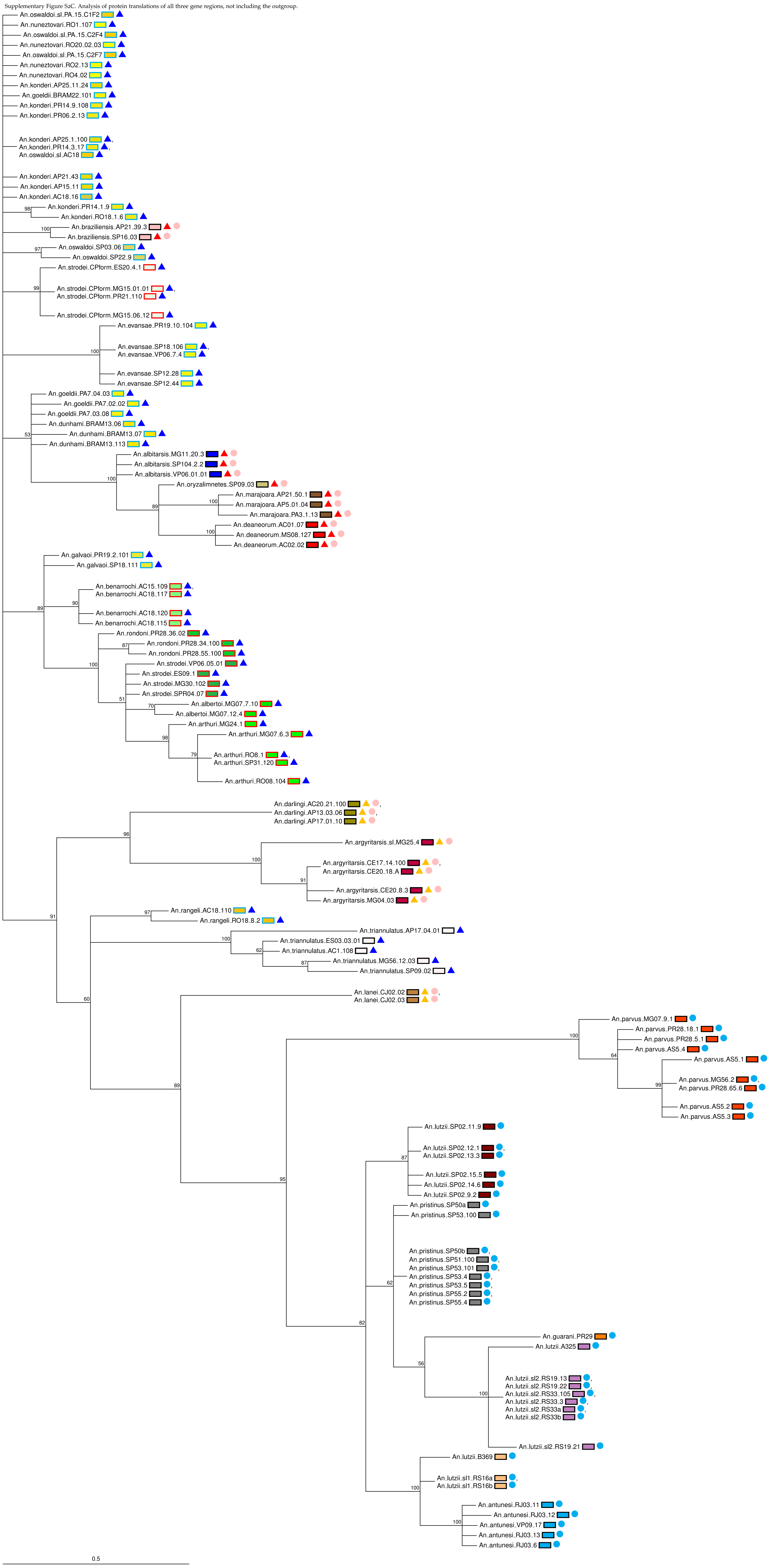

Supplement: Figure S2 — Phylogenetic trees from protein translations. Consensus trees of Bayesian analysis of translations of concatenated gene regions. See the caption in the Figure for details of the analysis. (A) Analysis of protein translations of all three gene regions, including An. kompi in the the outgroup. (B) Analysis of protein translations of all three gene regions, not including An. kompi in the the outgroup. (C) Analysis of protein translations of all three gene regions, not including the outgroup. (PDF) [file pone.0054063.s002.pdf]
