# Supplementary material for: Phylogenetic Analysis and DNA-based Species Confirmation in Anopheles (Nyssorhynchus)
Source: PLoS One. 2013 Feb 4;8(2):e54063. doi: 10.1371/journal.pone.0054063 (PMC3563636; doi:10.1371/journal.pone.0054063)
Supplement: Table S1 — Species and specimens. A table showing the specimens used in this study, and the morphologically-based species identification. (PDF) [file pone.0054063.s003.pdf]

Supplementary Table S1. Species and specimens used in this study

ALBIMANUS SECTION
OSWALDOI SERIES
Oswaldoi Group
Oswaldoi Subgroup
An. rangeli
3
An_rangeli_AC15_04
An_rangeli_AC18_110
An_rangeli_RO18_8_2
An. galvaoi
3
An_galvaoi_PR19_2_101
An_galvaoi_SP18_111
An_galvaoi_SP66_20_1
An. konderi A
4
An_konderi_AP15_11
An_konderi_AP21_43
An_konderi_AP25_11_24
An_konderi_AP25_1_100
An. konderi s. s.
5
An_konderi_PR06_2_13
An_konderi_PR14_1_9
An_konderi_PR14_3_17
An_konderi_PR14_9_108
An_konderi_RO18_1_6
An. konderi B
1
An_konderi_AC18_16
An. oswaldoi A
5
An_oswaldoi_sl_AC18_102
An_oswaldoi_sl_AC18_107
An_oswaldoi_sl_PA_15_C1F2
An_oswaldoi_sl_PA_15_C2F4
An_oswaldoi_sl_PA_15_C2F7
An. oswaldoi ss
3
An_oswaldoi_ES08_11_07
An_oswaldoi_SP03_06
An_oswaldoi_SP22_9
An. dunhami
3
An_dunhami_BRAM13_06
An_dunhami_BRAM13_07
An_dunhami_BRAM13_113
An. evansae
6
An_evansae_PR19_10_104
An_evansae_SP12_28
An_evansae_SP12_44
An_evansae_SP18_106
An_evansae_SP18_27
An_evansae_VP06_7_4
Nuneztovari Complex
An. nuneztovari
4
An_nuneztovari_RO1_107
An_nuneztovari_RO20_02_03
An_nuneztovari_RO2_13
An_nuneztovari_RO4_02
An. goeldii
6
An_goeldii_BRAM03_01
An_goeldii_BRAM22_101
An_goeldii_PA7_02_02
An_goeldii_PA7_03_08
An_goeldii_PA7_04_03
An_goeldii_PA7_17_02
Strodei Subgroup
An. strodei
4
An_strodei_ES09_1
An_strodei_MG30_102
An_strodei_SPR04_07
An_strodei_VP06_05_01
An. albertoi
2
An_albertoi_MG07_12_4
An_albertoi_MG07_7_10
An. strodei CPform
4
An_strodei_CPform_ES20_4_1
An_strodei_CPform_MG15_01_01
An_strodei_CPform_MG15_06_12
An_strodei_CPform_PR21_110
An. arthuri
5
An_arthuri_MG07_6_3
An_arthuri_MG24_1
An_arthuri_RO08_104
An_arthuri_RO8_1
An_arthuri_SP31_120
An. rondoni
3
An_rondoni_PR28_34_100
An_rondoni_PR28_36_02
An_rondoni_PR28_55_100
Benarrochi Complex
An. benarrochi
4
An_benarrochi_AC15_109
An_benarrochi_AC18_115
An_benarrochi_AC18_117
An_benarrochi_AC18_120
Triannulatus Group
An. triannulatus
5
An_triannulatus_AC1_108
An_triannulatus_AP17_04_01
An_triannulatus_ES03_03_01
An_triannulatus_MG56_12_03
An_triannulatus_SP09_02
ARGYRITARSIS SECTION
ALBITARSIS SERIES
Albitarsis Group
Albitarsis Complex
An. oryzalimnetes
1
An_oryzalimnetes_SP09_03
An. deaneorum
3
An_deaneorum_AC01_07

An_deaneorum_AC02_02
An_deaneorum_MS08_127
An. albitarsis
3
An_albitarsis_MG11_20_3
An_albitarsis_SP104_2_2
An_albitarsis_VP06_01_01
An. marajoara
3
An_marajoara_AP21_50_1
An_marajoara_AP5_01_04
An_marajoara_PA3_1_13
Braziliensis Group
An. braziliensis
2
An_braziliensis_AP21_39_3
An_braziliensis_SP16_03
ARGYRITARSIS SERIES
Argyritarsis Group
An. argyritarsis
4
An_argyritarsis_CE17_14_100
An_argyritarsis_CE20_18_A
An_argyritarsis_CE20_8_3
An_argyritarsis_MG04_03
An. argyritarsis s.l.
1
An_argyritarsis_sl_MG25_4
Darlingi Group
An. darlingi
3
An_darlingi_AC20_21_100
An_darlingi_AP13_03_06
An_darlingi_AP17_01_10
Lanei Group
An. lanei
2
An_lanei_CJ02_02
An_lanei_CJ02_03
Pictipennis Group
An. atacamensis
3
An_atacamensis_GQ902966
An_atacamensis_GQ902967
An_atacamensis_JF923686
MYZORHYNCHELLA SECTION
An. guarani
3
An_guarani_PR29
An_guarani_PR29_08
An_guarani_PR29_09_06
An. parvus
9
An_parvus_AS5_1
An_parvus_AS5_2
An_parvus_AS5_3
An_parvus_AS5_4
An_parvus_MG07_9_1
An_parvus_MG56_2
An_parvus_PR28_18_1
An_parvus_PR28_5_1
An_parvus_PR28_65_6
An. antunesi
6
An_antunesi_RJ03_11
An_antunesi_RJ03_12
An_antunesi_RJ03_13
An_antunesi_RJ03_6
An_antunesi_VP09_17
An_antunesi_VP11b
An. lutzii ss
7
An_lutzii_SP02_10_5
An_lutzii_SP02_11_9
An_lutzii_SP02_12_1
An_lutzii_SP02_13_3
An_lutzii_SP02_14_6
An_lutzii_SP02_15_5
An_lutzii_SP02_9_2
An. lutzii B
3
An_lutzii_B369
An_lutzii_sl1_RS16a
An_lutzii_sl1_RS16b
An. lutzii A
8
An_lutzii_A325
An_lutzii_sl2_RS19_13
An_lutzii_sl2_RS19_21
An_lutzii_sl2_RS19_22
An_lutzii_sl2_RS33_105
An_lutzii_sl2_RS33_3
An_lutzii_sl2_RS33a
An_lutzii_sl2_RS33b
An. pristinus
10
An_pristinus_SP50a
An_pristinus_SP50b
An_pristinus_SP51_100
An_pristinus_SP53_100
An_pristinus_SP53_101
An_pristinus_SP53_4
An_pristinus_SP53_5
An_pristinus_SP55_2
An_pristinus_SP55_4
An_pristinus_VP11a
OUTGROUP
An. kompi
1
An_kompi_SP69_22_5
An. cruzii
1
An_cruzii_ST16
An. intermedius
1
An_intermedius_SP22_106
