# Supplementary material for: Phylogenetic Analysis and DNA-based Species Confirmation in Anopheles (Nyssorhynchus)
Source: PLoS One. 2013 Feb 4;8(2):e54063. doi: 10.1371/journal.pone.0054063 (PMC3563636; doi:10.1371/journal.pone.0054063)
Supplement: Table S2 — Support for higher-level groupings from Bayesian analyses of DNA sequences. Support for species is shown in Table S6. Support is shown as Bayesian posterior probability as taken from the posterior distribution; that is from the sampled trees, not from a consensus tree. (PDF) [file pone.0054063.s004.pdf]

Supplementary Table S2A. Support for higher-level groupings from analyses of DNA sequences

		An konderi A + *konderi* B + *oswaldoi* A	*An konderi* A + *konderi* B + *oswaldoi* A + *konderi ss*	*An konderi* A + *konderi* B + *oswaldoi* A + *konderi ss* + *oswaldoi ss*	*An konderi* A + *konderi* B + *oswaldoi* A + *konderi ss* + *oswaldoi ss* + *evansae*	*An galvaoi* + *rangeli*	*An konderi* A + *konderi* B + *oswaldoi* A + *konderi ss* + *oswaldoi ss* + *evansae* + *galvaoi* + *rangeli*	Nunestovari Complex (*An nunestovari* + *goeldii*)	Nunestovari Complex + *dunhami*	Oswaldoi Subgroup	*An albertoi* + *strodei*	*An albertoi* + *strodei* + *arthuri*	*An albertoi* + *strodei* + *arthuri* + *rondoni*	*An albertoi* + *strodei* + *arthuri* + *rondoni* + CPForm	*An albertoi* + *strodei* + *arthuri* + *rondoni* + CPForm + *benarrochi*	Strodei Subgroup	Oswaldoi Series	Oswaldoi Series no *An benarrochi*	Oswaldoi Group
All 3 genes in 8 partitions, *An kompi* included	mb 1	1.0	0.996	1.0	0.652	1.0	0.580	1.0	1.0	0.892	1.0	0.764	1.0	1.0	0.052	0.052	0.0	0.0	1.0
	mb 2	0.992	1.0	1.0	0.648	1.0	0.612	1.0	1.0	0.876	1.0	0.864	1.0	0.996	0.060	0.060	0.0	0.0	1.0
	p4 0	0.988	0.966	0.012	0.0	1.0	0.0	0.998	1.0	0.0	1.0	0.632	1.0	1.0	0.042	0.042	0.0	0.0	0.0
	p4 1	0.996	1.0	1.0	0.632	1.0	0.622	0.998	1.0	0.892	1.0	0.600	1.0	0.998	0.078	0.078	0.0	0.0	0.998
	p4 2	1.0	0.996	1.0	0.632	1.0	0.602	1.0	1.0	0.908	1.0	0.654	1.0	1.0	0.060	0.060	0.0	0.0	0.990
	p4 3	0.998	1.0	1.0	0.702	1.0	0.588	0.998	1.0	0.940	1.0	0.578	1.0	1.0	0.072	0.072	0.0	0.0	1.0
	p4 4	0.998	1.0	1.0	0.614	1.0	0.626	1.0	1.0	0.898	1.0	0.868	1.0	1.0	0.062	0.062	0.0	0.0	1.0
All 3 genes in 8 partitions, *An kompi* excluded	mb 1	0.920	0.880	0.044	0.0	1.0	0.0	0.996	1.0	0.0	1.0	0.668	1.0	1.0	0.036	0.036	0.0	0.0	0.0
	mb 2	0.928	0.892	0.036	0.0	1.0	0.0	0.996	0.944	0.0	1.0	0.584	1.0	1.0	0.028	0.028	0.0	0.0	0.0
	p4 0	0.898	0.810	0.038	0.0	1.0	0.002	0.994	1.0	0.0	1.0	0.422	1.0	1.0	0.036	0.036	0.0	0.0	0.0
	p4 1	0.992	0.998	1.0	0.562	1.0	0.628	1.0	1.0	0.900	1.0	0.702	1.0	0.998	0.066	0.066	0.0	0.0	0.998
	p4 2	0.940	0.846	0.044	0.0	1.0	0.0	1.0	1.0	0.0	1.0	0.602	1.0	0.998	0.032	0.032	0.0	0.0	0.0
	p4 3	0.968	0.956	0.738	0.384	0.996	0.544	0.906	1.0	0.594	1.0	0.488	1.0	0.996	0.058	0.058	0.0	0.0	0.714
	p4 4	0.946	0.892	0.030	0.002	0.998	0.0	0.998	1.0	0.0	1.0	0.730	1.0	0.998	0.042	0.042	0.0	0.0	0.0
white gene only, in 3 partitions, *An kompi* excluded	mb 1	0.004	0.032	0.620	0.124	0.472	0.004	0.064	1.0	0.0	0.028	0.0	0.004	0.068	0.0	0.0	0.072	0.0	0.048
	mb 2	0.004	0.080	0.812	0.108	0.328	0.004	0.068	1.0	0.004	0.068	0.0	0.004	0.084	0.0	0.0	0.024	0.0	0.036
	p4 0	0.010	0.090	0.822	0.076	0.282	0.002	0.080	1.0	0.0	0.052	0.0	0.006	0.058	0.0	0.0	0.018	0.0	0.036
	p4 1	0.0	0.072	0.876	0.080	0.196	0.006	0.058	1.0	0.0	0.058	0.0	0.006	0.044	0.002	0.002	0.010	0.0	0.014
	p4 2	0.008	0.078	0.840	0.098	0.262	0.0	0.046	1.0	0.0	0.050	0.0	0.002	0.054	0.0	0.0	0.048	0.0	0.006
	p4 3	0.0	0.074	0.804	0.094	0.422	0.0	0.080	1.0	0.0	0.052	0.0	0.002	0.054	0.0	0.0	0.096	0.0	0.012
	p4 4	0.014	0.070	0.846	0.052	0.040	0.0	0.110	1.0	0.0	0.066	0.0	0.002	0.024	0.002	0.002	0.0	0.0	0.0
CAD gene only, in 3 partitions, *An kompi* excluded	mb 1	0.008	0.344	0.028	0.0	1.0	0.0	0.0	1.0	0.008	0.996	0.996	1.0	0.080	0.0	0.0	0.0	0.0	0.0
	mb 2	0.0	0.328	0.060	0.004	1.0	0.0	0.516	1.0	0.008	1.0	1.0	1.0	0.040	0.0	0.0	0.0	0.0	0.0
	p4 0	0.0	0.664	0.086	0.004	0.998	0.004	0.016	1.0	0.050	0.994	0.992	0.992	0.042	0.0	0.0	0.0	0.0	0.0
	p4 1	0.002	0.536	0.166	0.012	0.996	0.0	1.0	1.0	0.144	0.970	0.974	0.972	0.034	0.0	0.0	0.0	0.0	0.0
	p4 2	0.002	0.506	0.062	0.002	0.996	0.0	1.0	1.0	0.018	1.0	1.0	1.0	0.030	0.0	0.0	0.0	0.0	0.0
	p4 3	0.0	0.290	0.034	0.0	1.0	0.0	0.0	1.0	0.016	1.0	1.0	0.998	0.032	0.0	0.0	0.0	0.0	0.0
	p4 4	0.002	0.220	0.006	0.0	1.0	0.0	0.0	1.0	0.0	1.0	1.0	1.0	0.146	0.0	0.0	0.0	0.0	0.0
COI gene only, in 2 partitions, *An kompi* excluded	mb 1	0.464	0.0	0.0	0.036	0.100	0.056	1.0	1.0	0.0	0.760	0.0	1.0	0.884	0.060	0.060	0.0	0.0	0.776
	mb 2	0.488	0.0	0.0	0.028	0.052	0.044	1.0	1.0	0.0	0.768	0.0	1.0	0.992	0.088	0.088	0.012	0.0	0.752
	p4 0	0.474	0.0	0.0	0.026	0.092	0.046	0.994	1.0	0.0	0.780	0.0	0.994	0.996	0.098	0.098	0.002	0.0	0.756
	p4 1	0.478	0.0	0.0	0.040	0.098	0.054	0.998	1.0	0.0	0.814	0.0	1.0	0.996	0.088	0.088	0.004	0.0	0.750
	p4 2	0.492	0.0	0.0	0.046	0.096	0.052	0.994	1.0	0.0	0.840	0.0	0.998	0.986	0.090	0.090	0.016	0.0	0.728
	p4 3	0.472	0.0	0.0	0.042	0.088	0.050	1.0	0.994	0.0	0.784	0.0	0.998	0.994	0.122	0.122	0.002	0.0	0.660
	p4 4	0.410	0.0	0.0	0.036	0.056	0.062	1.0	0.994	0.0	0.778	0.0	1.0	0.994	0.086	0.086	0.004	0.0	0.758

Supplementary Table S2B. Support for higher-level groupings from analyses of DNA sequences

		Oswaldoi Group no *An benarrochi*	Oswaldoi Group with *An braziliensis*	Oswaldoi Group no *An benarrochi* with *An braziliensis*	*An triannulatus + darlingi*	*An triannulatus + rangeli*	*An triannulatus + argyritarsis*	*An triannulatus + lanei*	*An argyritarsis + darlingi*	*An argyritarsis + lanei + darlingi*	Argyritarsis Series	Argyritarsis Series + *An triannulatus*	Albitarsis Group/Complex	Albitarsis Series	Argyritarsis Section	Argyritarsis Section + *An triannulatus*	*An antunesi + lutzii* B	*An lutzii ss + lutzii* A	Myzorhynchella Crown (no *An parvus*)	Myzorhynchella Section	outgroup
All 3 genes in 8 partitions, *An kompi* included	mb 1	0.856	0.620	0.856	0.980	0.0	0.0	0.0	0.008	0.0	0.0	0.0	1.0	0.004	0.0	0.0	1.0	0.944	1.0	0.520	0.0
	mb 2	0.860	0.684	0.860	0.972	0.0	0.0	0.0	0.0	0.0	0.0	0.0	1.0	0.0	0.0	0.0	1.0	0.940	1.0	0.556	0.0
	p4 0	0.0	0.0	0.0	0.986	0.0	0.0	0.0	0.0	0.0	0.0	0.0	1.0	0.0	0.0	0.0	1.0	0.958	0.962	0.656	0.0
	p4 1	0.836	0.732	0.836	0.982	0.0	0.0	0.0	0.0	0.0	0.0	0.0	1.0	0.002	0.0	0.0	1.0	0.960	1.0	0.696	0.0
	p4 2	0.862	0.642	0.862	0.974	0.0	0.0	0.0	0.0	0.0	0.0	0.0	1.0	0.004	0.0	0.0	1.0	0.948	1.0	0.714	0.0
	p4 3	0.838	0.678	0.838	0.988	0.0	0.0	0.0	0.0	0.0	0.0	0.0	1.0	0.0	0.0	0.0	1.0	0.940	1.0	0.676	0.0
	p4 4	0.848	0.692	0.848	0.974	0.0	0.0	0.0	0.0	0.0	0.0	0.0	1.0	0.0	0.0	0.0	1.0	0.946	1.0	0.702	0.0
All 3 genes in 8 partitions, *An kompi* excluded	mb 1	0.0	0.0	0.0	0.952	0.0	0.0	0.0	0.0	0.0	0.0	0.0	1.0	0.004	0.0	0.0	1.0	0.964	1.0	1.0	0.820
	mb 2	0.0	0.0	0.0	0.956	0.0	0.0	0.0	0.0	0.0	0.0	0.0	1.0	0.004	0.0	0.0	1.0	0.952	0.720	0.704	1.0
	p4 0	0.0	0.0	0.0	0.960	0.0	0.0	0.0	0.0	0.0	0.0	0.0	1.0	0.004	0.0	0.0	1.0	0.976	1.0	0.996	0.644
	p4 1	0.854	0.608	0.854	0.948	0.0	0.0	0.0	0.0	0.0	0.0	0.0	1.0	0.0	0.0	0.0	1.0	0.964	1.0	0.996	0.968
	p4 2	0.0	0.0	0.0	0.970	0.0	0.0	0.0	0.0	0.0	0.0	0.0	1.0	0.010	0.0	0.0	1.0	0.958	0.870	0.862	1.0
	p4 3	0.592	0.404	0.592	0.956	0.0	0.0	0.0	0.0	0.0	0.0	0.0	1.0	0.002	0.0	0.0	1.0	0.962	0.944	0.936	0.978
	p4 4	0.0	0.0	0.0	0.990	0.0	0.0	0.0	0.0	0.0	0.0	0.0	0.996	0.0	0.0	0.0	1.0	0.962	0.806	0.788	1.0
white gene only, in 3 partitions, *An kompi* excluded	mb 1	0.0	0.0	0.0	0.144	0.004	0.012	0.0	0.008	0.0	0.0	0.0	0.012	0.0	0.0	0.0	0.988	1.0	1.0	0.096	1.0
	mb 2	0.0	0.004	0.0	0.244	0.004	0.0	0.0	0.016	0.0	0.0	0.0	0.008	0.0	0.0	0.0	0.984	1.0	1.0	0.088	1.0
	p4 0	0.0	0.0	0.0	0.170	0.008	0.034	0.0	0.044	0.0	0.0	0.0	0.006	0.0	0.0	0.0	0.990	1.0	1.0	0.088	1.0
	p4 1	0.0	0.0	0.0	0.276	0.008	0.004	0.0	0.018	0.0	0.0	0.0	0.004	0.0	0.0	0.0	0.994	1.0	1.0	0.080	1.0
	p4 2	0.0	0.0	0.0	0.224	0.010	0.004	0.0	0.046	0.0	0.0	0.0	0.010	0.0	0.0	0.0	0.996	1.0	1.0	0.074	1.0
	p4 3	0.0	0.0	0.0	0.230	0.014	0.010	0.0	0.014	0.0	0.0	0.0	0.012	0.0	0.0	0.0	0.994	1.0	1.0	0.102	1.0
	p4 4	0.0	0.0	0.0	0.330	0.0	0.002	0.0	0.014	0.0	0.0	0.0	0.008	0.0	0.0	0.0	0.996	1.0	1.0	0.122	1.0
CAD gene only, in 3 partitions, *An kompi* excluded	mb 1	0.004	0.0	0.004	0.004	0.0	0.296	0.0	0.0	0.0	0.0	0.0	1.0	0.064	0.0	0.0	1.0	0.312	1.0	0.024	1.0
	mb 2	0.0	0.0	0.0	0.024	0.0	0.276	0.0	0.004	0.0	0.0	0.0	1.0	0.092	0.0	0.0	1.0	0.360	1.0	0.060	1.0
	p4 0	0.0	0.0	0.0	0.0	0.0	0.042	0.004	0.0	0.0	0.0	0.0	1.0	0.054	0.0	0.0	1.0	0.438	1.0	0.0	0.0
	p4 1	0.032	0.008	0.032	0.0	0.0	0.324	0.0	0.0	0.0	0.0	0.0	1.0	0.106	0.0	0.0	1.0	0.386	1.0	0.0	1.0
	p4 2	0.002	0.0	0.002	0.0	0.0	0.122	0.0	0.0	0.0	0.0	0.0	1.0	0.130	0.0	0.0	1.0	0.384	1.0	0.0	0.0
	p4 3	0.006	0.0	0.006	0.0	0.0	0.304	0.0	0.0	0.0	0.0	0.0	1.0	0.080	0.0	0.0	1.0	0.360	1.0	0.0	1.0
	p4 4	0.0	0.0	0.0	0.446	0.0	0.048	0.0	0.004	0.0	0.0	0.0	1.0	0.010	0.0	0.0	1.0	0.260	1.0	0.828	0.0
COI gene only, in 2 partitions, *An kompi* excluded	mb 1	0.0	0.0	0.0	0.064	0.0	0.012	0.004	0.016	0.576	0.0	0.0	0.936	0.0	0.0	0.0	0.484	0.004	0.0	0.0	0.0
	mb 2	0.0	0.0	0.0	0.012	0.0	0.0	0.004	0.008	0.324	0.0	0.0	0.952	0.0	0.0	0.0	0.500	0.0	0.0	0.0	0.0
	p4 0	0.0	0.002	0.0	0.038	0.002	0.004	0.004	0.008	0.298	0.0	0.010	0.962	0.0	0.0	0.0	0.556	0.0	0.012	0.0	0.012
	p4 1	0.002	0.002	0.002	0.008	0.0	0.004	0.002	0.046	0.642	0.0	0.0	0.964	0.0	0.0	0.0	0.584	0.0	0.0	0.0	0.004
	p4 2	0.0	0.0	0.0	0.004	0.0	0.002	0.0	0.028	0.626	0.0	0.0	0.920	0.002	0.0	0.0	0.614	0.0	0.0	0.0	0.0
	p4 3	0.0	0.0	0.0	0.004	0.0	0.002	0.004	0.020	0.426	0.0	0.0	0.954	0.004	0.0	0.0	0.514	0.0	0.0	0.0	0.008
	p4 4	0.002	0.002	0.002	0.010	0.0	0.006	0.0	0.022	0.560	0.0	0.0	0.902	0.002	0.0	0.0	0.568	0.004	0.0	0.0	0.0
