# Supplementary material for: Phylogenetic Analysis and DNA-based Species Confirmation in Anopheles (Nyssorhynchus)
Source: PLoS One. 2013 Feb 4;8(2):e54063. doi: 10.1371/journal.pone.0054063 (PMC3563636; doi:10.1371/journal.pone.0054063)
Supplement: Table S3 — Support for species and higher-order groupings from gene translations. Support is shown as Bayesian posterior probability as taken from the posterior distribution; that is from the sampled trees, not from a consensus tree. (PDF) [file pone.0054063.s005.pdf]

Supplementary Table S3A. Support for species and higher-order groupings from gene translations

		An albitarsis	*An antunesi*	*An argyritarsis*	*An arthuri*	*An benarrochi*	*An braziliensis*	*An deaneorum*	*An dunhami*	*An evansae*	*An galvaoi*	*An goeldii*	*An konderi* A and ss *An oswaldoi* A	*An lutzii* B	*An lutzii* A	*An lutzii ss*	*An marajoara*	*An nuneztovari*	*An oswaldoi ss*	*An parvus*	*An pristinus*	*An rangeli*	*An rondoni*	*An strodei*	*An strodei* CPform	*An triannulatus*
An kompi included	mb 1	0.264	1.0	0.942	0.976	0.976	0.998	1.0	0.042	1.0	0.452	0.0	0.0	0.346	1.0	0.978	1.0	0.036	0.958	0.996	0.242	0.990	0.294	0.018	0.980	0.996
	mb 2	0.276	0.998	0.954	0.982	0.958	1.0	1.0	0.034	1.0	0.524	0.0	0.0	0.328	0.996	0.978	1.0	0.034	0.974	0.992	0.280	0.994	0.310	0.018	0.992	0.998
	p4 0	0.284	1.0	0.988	0.982	0.932	0.998	0.996	0.050	0.996	0.610	0.0	0.0	0.502	1.0	0.968	0.990	0.026	0.964	1.0	0.264	0.988	0.302	0.048	0.994	0.996
	p4 1	0.400	1.0	1.0	0.978	0.960	1.0	0.998	0.032	1.0	0.536	0.0	0.0	0.518	1.0	0.950	1.0	0.024	0.974	1.0	0.338	0.994	0.292	0.044	0.974	1.0
	p4 2	0.376	0.998	1.0	0.986	0.964	1.0	0.998	0.038	1.0	0.564	0.0	0.0	0.482	1.0	0.956	0.998	0.038	0.974	1.0	0.276	0.998	0.266	0.052	0.996	1.0
	p4 3	0.260	1.0	0.952	0.984	0.976	0.998	1.0	0.024	0.998	0.564	0.0	0.0	0.490	1.0	0.970	1.0	0.028	0.976	0.978	0.278	0.988	0.284	0.042	0.962	0.996
	p4 4	0.272	1.0	0.984	0.978	0.958	0.998	0.998	0.032	1.0	0.536	0.0	0.0	0.464	0.998	0.950	1.0	0.038	0.968	1.0	0.292	0.988	0.270	0.030	0.992	1.0
	p4 5	0.316	0.996	0.984	0.996	0.948	0.996	0.994	0.048	0.998	0.550	0.0	0.0	0.482	1.0	0.974	0.986	0.040	0.958	0.992	0.304	0.994	0.264	0.038	0.996	0.978
	p4 6	0.436	1.0	0.966	0.990	0.946	0.994	0.996	0.036	0.998	0.532	0.0	0.0	0.490	0.998	0.970	1.0	0.034	0.974	0.990	0.260	0.992	0.252	0.034	0.984	0.992
	p4 7	0.346	1.0	1.0	0.982	0.948	0.996	0.998	0.044	0.994	0.522	0.0	0.0	0.486	0.998	0.964	1.0	0.042	0.978	0.988	0.300	0.990	0.302	0.042	0.964	1.0
	p4 8	0.314	1.0	0.942	0.986	0.966	0.996	1.0	0.024	0.998	0.554	0.0	0.0	0.490	1.0	0.970	1.0	0.050	0.984	0.992	0.302	0.984	0.298	0.042	0.974	0.986
	p4 9	0.340	1.0	1.0	0.982	0.960	1.0	0.998	0.030	1.0	0.544	0.0	0.0	0.496	0.998	0.968	1.0	0.050	0.968	0.996	0.332	0.988	0.276	0.042	0.980	1.0
An kompi excluded	mb 1	0.236	1.0	1.0	0.984	0.940	1.0	1.0	0.044	1.0	0.584	0.0	0.0	0.336	0.996	0.964	1.0	0.064	0.976	1.0	0.292	0.988	0.308	0.016	0.932	0.996
	mb 2	0.236	1.0	1.0	0.968	0.968	1.0	1.0	0.020	1.0	0.508	0.0	0.0	0.352	1.0	0.972	1.0	0.036	0.960	1.0	0.212	1.0	0.296	0.032	0.844	1.0
	p4 0	0.416	1.0	1.0	0.983	0.844	0.998	0.995	0.022	1.0	0.201	0.0	0.0	0.088	1.0	0.865	1.0	0.013	0.867	1.0	0.132	0.949	0.091	0.010	0.861	1.0
	p4 1	0.086	1.0	1.0	0.998	0.849	0.998	0.999	0.021	1.0	0.235	0.0	0.0	0.110	0.999	0.859	1.0	0.021	0.899	1.0	0.146	0.980	0.120	0.014	0.964	1.0
	p4 2	0.206	0.998	1.0	0.983	0.826	0.999	0.988	0.012	1.0	0.281	0.0	0.0	0.102	0.996	0.892	1.0	0.010	0.934	1.0	0.119	0.979	0.123	0.010	0.900	1.0
	p4 3	0.173	1.0	1.0	0.976	0.849	0.997	1.0	0.011	1.0	0.194	0.0	0.0	0.089	0.996	0.889	1.0	0.018	0.845	1.0	0.101	0.963	0.097	0.008	0.619	0.996
	p4 4	0.108	1.0	1.0	0.985	0.881	0.997	0.996	0.012	1.0	0.225	0.0	0.0	0.106	0.999	0.855	1.0	0.030	0.937	1.0	0.118	0.966	0.103	0.011	0.967	1.0
No outgroup	mb 1	0.288	1.0	1.0	0.966	0.970	1.0	1.0	0.050	1.0	0.618	0.0	0.0	0.316	0.998	0.966	1.0	0.034	0.982	1.0	0.230	0.996	0.300	0.020	1.0	1.0
	mb 2	0.408	1.0	1.0	0.964	0.976	1.0	1.0	0.034	1.0	0.546	0.0	0.0	0.330	0.998	0.978	1.0	0.036	0.984	1.0	0.272	0.998	0.308	0.016	0.998	1.0
	p4 0	0.151	1.0	1.0	0.953	0.889	0.998	1.0	0.020	1.0	0.265	0.0	0.0	0.119	0.992	0.862	1.0	0.019	0.962	1.0	0.124	0.974	0.083	0.022	0.975	1.0
	p4 1	0.236	1.0	1.0	0.986	0.867	1.0	0.999	0.021	1.0	0.297	0.0	0.0	0.119	0.995	0.835	1.0	0.015	0.961	1.0	0.150	0.980	0.133	0.019	0.988	1.0
	p4 2	0.305	1.0	1.0	0.990	0.901	1.0	1.0	0.017	1.0	0.243	0.0	0.0	0.109	0.995	0.850	1.0	0.016	0.978	1.0	0.146	0.961	0.111	0.017	0.988	1.0
	p4 3	0.212	1.0	1.0	0.989	0.900	0.999	1.0	0.015	1.0	0.271	0.0	0.0	0.106	1.0	0.846	1.0	0.019	0.958	1.0	0.121	0.944	0.122	0.018	0.992	1.0
	p4 4	0.167	0.999	1.0	0.988	0.876	1.0	1.0	0.013	1.0	0.239	0.0	0.0	0.112	0.998	0.868	1.0	0.029	0.967	1.0	0.126	0.985	0.126	0.012	0.985	1.0

Supplementary Table S3B. Support for species and higher-order groupings from gene translations

		An konderi A + *ss* + *An oswaldoi* A+ *An konderi* B	*An konderi* A + *ss* + *oswaldoi* A + *konderi* B + *oswaldoi ss*	*An konderi* A and *ss* + *oswaldoi* A + *konderi* B + *oswaldoi ss* + *evansae*	*An galvaoi* + *rangeli*	*An konderi* A + *ss* + *oswaldoi* A + *oswaldoi ss* + *konderi* B + *evansae* + *galvaoi* + *rangeli*	Nunestovari Complex (*nunestovari* + *goeldii*)	Nunestovari Complex + *dunhami*	Oswaldoi Subgroup	*An albertoi* + *strodei*	*An albertoi* + *strodei* + *arthuri*	*An albertoi* + *strodei* + *arthuri* + *rondoni*	*An albertoi* + *strodei* + *arthuri* + *rondoni* + CPForm	*An albertoi* + *strodei* + *arthuri* + *rondoni* + CPForm + *benarrochi*	Strodei Subgroup	Oswaldoi Series	Oswaldoi Series (no *An benarrochi*)	Oswaldoi Group	Oswaldoi Group (no *An benarrochi*)	Oswaldoi Group with *An braziliensis*	Oswaldoi Group without *An benarrochi*, with *An braziliensis*
An kompi included	mb 1	0.002	0.006	0.004	0.0	0.0	0.0	0.008	0.0	0.068	0.566	0.996	0.0	0.0	0.0	0.0	0.0	0.0	0.0	0.0	0.0
	mb 2	0.004	0.006	0.006	0.004	0.0	0.0	0.020	0.0	0.062	0.612	1.0	0.002	0.0	0.0	0.0	0.0	0.0	0.0	0.004	0.0
	p4 0	0.006	0.006	0.004	0.0	0.0	0.0	0.018	0.0	0.054	0.576	1.0	0.0	0.0	0.0	0.0	0.0	0.0	0.0	0.0	0.0
	p4 1	0.006	0.0	0.002	0.0	0.0	0.0	0.028	0.0	0.062	0.578	0.984	0.0	0.0	0.0	0.0	0.0	0.0	0.0	0.0	0.0
	p4 2	0.006	0.008	0.018	0.0	0.0	0.002	0.044	0.0	0.112	0.614	0.994	0.0	0.0	0.0	0.0	0.0	0.0	0.0	0.002	0.0
	p4 3	0.006	0.006	0.010	0.0	0.0	0.002	0.010	0.0	0.084	0.586	1.0	0.0	0.0	0.0	0.0	0.0	0.0	0.0	0.0	0.0
	p4 4	0.0	0.004	0.004	0.0	0.0	0.0	0.014	0.0	0.066	0.558	1.0	0.0	0.0	0.0	0.0	0.0	0.0	0.0	0.0	0.0
	p4 5	0.004	0.010	0.014	0.0	0.0	0.0	0.022	0.0	0.092	0.624	0.986	0.0	0.0	0.0	0.0	0.0	0.0	0.0	0.004	0.0
	p4 6	0.008	0.002	0.012	0.0	0.0	0.0	0.060	0.0	0.092	0.550	0.990	0.0	0.0	0.0	0.0	0.0	0.0	0.0	0.010	0.0
	p4 7	0.004	0.006	0.014	0.0	0.0	0.0	0.008	0.0	0.088	0.562	0.998	0.0	0.0	0.0	0.0	0.0	0.0	0.0	0.0	0.0
	p4 8	0.010	0.002	0.012	0.0	0.0	0.0	0.014	0.0	0.106	0.580	0.996	0.0	0.0	0.0	0.0	0.0	0.0	0.0	0.0	0.0
	p4 9	0.006	0.002	0.004	0.0	0.0	0.0	0.022	0.0	0.068	0.608	0.994	0.0	0.0	0.0	0.0	0.0	0.004	0.0	0.006	0.0
An kompi excluded	mb 1	0.004	0.0	0.008	0.0	0.0	0.0	0.004	0.0	0.056	0.604	0.996	0.0	0.0	0.0	0.0	0.0	0.0	0.0	0.0	0.0
	mb 2	0.008	0.0	0.016	0.0	0.0	0.0	0.016	0.0	0.080	0.548	1.0	0.0	0.0	0.0	0.0	0.0	0.0	0.0	0.0	0.0
	p4 0	0.001	0.0	0.003	0.0	0.0	0.0	0.057	0.0	0.026	0.516	0.987	0.0	0.0	0.0	0.0	0.0	0.001	0.0	0.007	0.0
	p4 1	0.007	0.002	0.004	0.0	0.0	0.0	0.001	0.0	0.033	0.470	0.994	0.001	0.0	0.0	0.0	0.0	0.0	0.0	0.0	0.0
	p4 2	0.001	0.001	0.0	0.0	0.0	0.0	0.0	0.0	0.033	0.425	1.0	0.002	0.0	0.0	0.0	0.0	0.0	0.0	0.004	0.0
	p4 3	0.005	0.002	0.0	0.0	0.0	0.0	0.002	0.0	0.034	0.526	0.997	0.0	0.0	0.0	0.0	0.0	0.0	0.0	0.0	0.0
	p4 4	0.0	0.0	0.0	0.0	0.0	0.0	0.002	0.0	0.035	0.451	0.942	0.0	0.0	0.0	0.0	0.0	0.0	0.0	0.0	0.0
No outgroup	mb 1	0.002	0.002	0.004	0.0	0.0	0.0	0.038	0.0	0.068	0.614	1.0	0.0	0.0	0.0	0.0	0.0	0.0	0.0	0.006	0.0
	mb 2	0.0	0.006	0.006	0.0	0.0	0.0	0.096	0.0	0.054	0.570	1.0	0.002	0.0	0.0	0.0	0.0	0.0	0.0	0.002	0.0
	p4 0	0.001	0.001	0.003	0.0	0.0	0.0	0.004	0.0	0.033	0.473	1.0	0.0	0.0	0.0	0.0	0.0	0.0	0.0	0.001	0.0
	p4 1	0.003	0.0	0.006	0.0	0.0	0.0	0.006	0.0	0.035	0.447	1.0	0.0	0.0	0.0	0.0	0.0	0.0	0.0	0.001	0.0
	p4 2	0.001	0.004	0.0	0.0	0.0	0.0	0.021	0.0	0.029	0.535	1.0	0.0	0.0	0.0	0.0	0.0	0.0	0.0	0.001	0.0
	p4 3	0.002	0.0	0.002	0.0	0.0	0.0	0.020	0.0	0.039	0.527	1.0	0.0	0.0	0.0	0.0	0.0	0.0	0.0	0.001	0.0
	p4 4	0.001	0.002	0.007	0.0	0.0	0.0	0.006	0.0	0.038	0.481	0.999	0.0	0.0	0.0	0.0	0.0	0.0	0.0	0.0	0.0

Supplementary Table S3C. Support for species and higher-order groupings from gene translations

		An triannulatus + darlingi	*An triannulatus + rangeli*	*An triannulatus + argyritarsis*	*An triannulatus + lanei*	*An argyritarsis + darlingi*	*An argyritarsis + lanei + darlingi*	Argyritarsis Series	Argyritarsis Series + *An triannulatus*	Albitarsis Group/Complex	Albitarsis Series	Argyritarsis Section	Argyritarsis Section + *An triannulatus*	*An antunesi + lutzii* B	*An lutzii ss + lutzii* A	Myzorhynchella Crown (no *An parvus*)	Myzorhynchella Section	outgroup
An kompi included	mb 1	0.002	0.284	0.0	0.014	0.416	0.0	0.0	0.0	0.760	0.002	0.0	0.0	1.0	0.062	0.958	0.874	0.354
	mb 2	0.0	0.276	0.0	0.004	0.570	0.002	0.002	0.0	0.690	0.0	0.0	0.0	1.0	0.050	0.968	0.462	0.038
	p4 0	0.0	0.368	0.0	0.018	0.742	0.0	0.0	0.0	0.680	0.0	0.0	0.0	1.0	0.058	0.936	0.966	0.784
	p4 1	0.0	0.396	0.0	0.012	0.852	0.0	0.0	0.0	0.840	0.012	0.0	0.0	0.998	0.054	0.934	0.922	0.988
	p4 2	0.0	0.398	0.0	0.038	0.950	0.004	0.004	0.0	0.608	0.002	0.0	0.0	0.998	0.066	0.952	0.948	0.974
	p4 3	0.006	0.270	0.0	0.042	0.658	0.0	0.0	0.0	0.698	0.0	0.0	0.0	1.0	0.062	0.982	0.352	0.0
	p4 4	0.0	0.320	0.0	0.046	0.876	0.0	0.0	0.0	0.640	0.0	0.0	0.0	0.998	0.040	0.936	0.946	0.882
	p4 5	0.002	0.410	0.0	0.024	0.698	0.0	0.0	0.0	0.724	0.0	0.0	0.0	1.0	0.052	0.974	0.768	0.484
	p4 6	0.002	0.406	0.0	0.024	0.582	0.002	0.002	0.0	0.842	0.002	0.0	0.0	0.996	0.054	0.958	0.748	0.458
	p4 7	0.0	0.388	0.0	0.026	0.972	0.0	0.0	0.0	0.770	0.0	0.0	0.0	0.996	0.036	0.932	0.600	0.588
	p4 8	0.002	0.382	0.0	0.024	0.386	0.0	0.0	0.0	0.770	0.0	0.0	0.0	0.996	0.062	0.972	0.634	0.038
	p4 9	0.0	0.406	0.0	0.024	0.876	0.004	0.004	0.0	0.822	0.0	0.0	0.0	0.998	0.056	0.962	0.896	0.944
An kompi excluded	mb 1	0.0	0.372	0.0	0.004	0.980	0.0	0.0	0.0	0.772	0.0	0.0	0.0	1.0	0.040	0.916	0.940	0.840
	mb 2	0.0	0.308	0.0	0.004	0.976	0.0	0.0	0.0	0.864	0.0	0.0	0.0	1.0	0.044	0.956	0.948	0.632
	p4 0	0.0	0.449	0.0	0.011	0.853	0.0	0.0	0.0	0.821	0.001	0.0	0.0	1.0	0.029	0.819	0.936	1.0
	p4 1	0.001	0.261	0.0	0.029	0.916	0.001	0.001	0.0	0.538	0.0	0.0	0.0	0.999	0.021	0.839	0.950	1.0
	p4 2	0.001	0.274	0.0	0.032	0.939	0.0	0.0	0.0	0.697	0.0	0.0	0.0	0.999	0.027	0.876	0.953	1.0
	p4 3	0.001	0.506	0.0	0.001	0.828	0.0	0.0	0.0	0.988	0.0	0.0	0.0	1.0	0.021	0.838	0.766	0.0
	p4 4	0.0	0.313	0.0	0.010	0.935	0.001	0.001	0.0	0.663	0.0	0.0	0.0	1.0	0.034	0.840	0.956	1.0
No outgroup	mb 1	0.0	0.388	0.0	0.002	0.994	0.0	0.0	0.0	1.0	0.0	0.0	0.0	1.0	0.028	0.932	0.972	na
	mb 2	0.0	0.454	0.0	0.006	0.990	0.0	0.0	0.0	1.0	0.0	0.0	0.0	1.0	0.028	0.944	0.960	na
	p4 0	0.0	0.392	0.0	0.023	0.953	0.002	0.002	0.0	1.0	0.0	0.0	0.0	1.0	0.019	0.814	0.940	na
	p4 1	0.0	0.438	0.0	0.015	0.970	0.002	0.002	0.0	1.0	0.0	0.0	0.0	1.0	0.018	0.785	0.937	na
	p4 2	0.0	0.506	0.0	0.007	0.948	0.0	0.0	0.0	1.0	0.001	0.0	0.0	1.0	0.028	0.788	0.956	na
	p4 3	0.0	0.491	0.0	0.018	0.934	0.001	0.001	0.001	1.0	0.0	0.0	0.0	1.0	0.023	0.765	0.942	na
	p4 4	0.0	0.389	0.0	0.020	0.952	0.0	0.0	0.0	1.0	0.0	0.0	0.0	0.999	0.027	0.836	0.957	na
