# Supplementary material for: Phylogenetic Analysis and DNA-based Species Confirmation in Anopheles (Nyssorhynchus)
Source: PLoS One. 2013 Feb 4;8(2):e54063. doi: 10.1371/journal.pone.0054063 (PMC3563636; doi:10.1371/journal.pone.0054063)
Supplement: Table S4 — Support for species from three concatenated gene regions and from single gene regions only. Support is shown as Bayesian posterior probability as taken from the posterior distribution; that is from the sampled trees, not from a consensus tree. Support for higher-level groupings is shown in Table S4. (PDF) [file pone.0054063.s006.pdf]

Supplementary Table S4. Support for species from three concatenated genes and from single genes only

species	mb 1	mb 2	p4 0	p4 1	p4 2	p4 3	p4 4
Analysis of all three genes, in 8 data partitions							
An albertoi	1.0	1.0	1.0	1.0	1.0	1.0	1.0
An albitarsis	1.0	1.0	1.0	1.0	1.0	1.0	1.0
An antunesi	1.0	1.0	1.0	1.0	1.0	1.0	1.0
An argyritarsis	1.0	1.0	1.0	1.0	1.0	1.0	1.0
An arthuri	1.0	1.0	1.0	1.0	1.0	1.0	1.0
An atacamensis	1.0	1.0	1.0	1.0	1.0	1.0	1.0
An benarrochi	1.0	1.0	1.0	1.0	1.0	1.0	1.0
An braziliensis	1.0	1.0	1.0	1.0	1.0	1.0	1.0
An darlingi	1.0	1.0	1.0	1.0	1.0	1.0	1.0
An deaneorum	1.0	1.0	1.0	1.0	1.0	1.0	1.0
An dunhami	1.0	0.956	1.0	1.0	1.0	1.0	1.0
An evansae	1.0	1.0	1.0	1.0	1.0	1.0	1.0
An galvaoi	1.0	1.0	1.0	1.0	1.0	1.0	1.0
An goeldii	0.704	0.708	0.790	0.768	0.690	0.600	0.774
An guarani	1.0	1.0	1.0	1.0	1.0	1.0	1.0
An konderi ss	1.0	0.984	0.994	1.0	0.980	0.998	0.994
An konderi A	0.992	1.0	0.998	1.0	0.996	0.990	0.996
An lanei	1.0	1.0	1.0	1.0	1.0	1.0	1.0
An lutzii B	0.720	0.664	0.656	0.664	0.686	0.714	0.732
An lutzii A	1.0	1.0	1.0	1.0	1.0	1.0	1.0
An lutzii ss	1.0	1.0	1.0	1.0	1.0	1.0	1.0
An marajoara	1.0	1.0	1.0	1.0	1.0	1.0	1.0
An nuneztovari	0.996	0.980	0.970	0.990	0.992	0.998	0.988
An oswaldoi A	0.996	1.0	0.996	1.0	0.998	1.0	0.996
An oswaldoi ss	0.720	0.664	0.800	1.0	0.668	0.902	0.642
An parvus	1.0	1.0	1.0	1.0	1.0	1.0	1.0
An pristinus	1.0	1.0	1.0	1.0	1.0	1.0	1.0
An rangeli	1.0	1.0	1.0	1.0	1.0	1.0	1.0
An rondoni	1.0	1.0	1.0	1.0	1.0	1.0	1.0
An strodei	1.0	0.996	0.922	0.828	0.998	0.906	0.380
An strodei CPform	1.0	1.0	1.0	1.0	1.0	1.0	1.0
An triannulatus	1.0	1.0	1.0	1.0	1.0	1.0	1.0
Analysis of the *white* gene only, in 3 data partions							
An albitarsis	0.996	0.992	0.992	0.992	0.990	0.990	0.990
An antunesi	0.980	0.980	0.974	0.980	0.974	0.982	0.976
An argyritarsis	1.0	1.0	1.0	1.0	1.0	1.0	1.0
An arthuri	0.612	0.604	0.610	0.664	0.630	0.634	0.586
An benarrochi	1.0	0.996	0.998	0.996	1.0	1.0	1.0
An braziliensis	1.0	1.0	1.0	1.0	1.0	1.0	1.0
An darlingi	1.0	1.0	0.996	1.0	1.0	0.996	1.0
An deaneorum	1.0	1.0	1.0	1.0	1.0	1.0	1.0
An dunhami	0.180	0.180	0.220	0.236	0.254	0.238	0.262
An evansae	0.780	0.784	0.730	0.774	0.832	0.826	0.764
An galvaoi	0.996	0.984	0.992	0.992	0.996	0.996	0.996
An goeldii	0.364	0.376	0.302	0.390	0.300	0.398	0.380
An konderi ss	0.016	0.024	0.010	0.014	0.018	0.006	0.016
An konderi A	0.024	0.064	0.078	0.104	0.066	0.072	0.088
An lutzii B	1.0	1.0	0.998	0.998	0.996	0.998	0.998
An lutzii A	1.0	1.0	1.0	1.0	1.0	1.0	1.0
An lutzii ss	0.996	0.992	0.994	0.990	0.992	0.992	0.996
An marajoara	0.020	0.040	0.034	0.030	0.032	0.062	0.032
An nuneztovari	0.988	0.992	0.996	0.986	0.998	0.992	0.994
An oswaldoi A	0.132	0.096	0.124	0.132	0.138	0.164	0.152
An oswaldoi ss	0.908	0.880	0.924	0.898	0.884	0.886	0.900
An parvus	1.0	1.0	1.0	1.0	1.0	1.0	1.0
An pristinus	0.992	0.964	0.976	0.978	0.992	0.980	0.982
An rangeli	0.980	0.976	0.958	0.942	0.950	0.964	0.928
An rondoni	1.0	0.988	0.980	0.984	0.994	0.994	0.996
An strodei	0.004	0.024	0.008	0.018	0.006	0.010	0.010
An strodei CPform	0.996	1.0	0.998	0.990	0.998	0.998	1.0
An triannulatus	1.0	1.0	1.0	1.0	1.0	1.0	1.0

species	mb 1	mb 2	p4 0	p4 1	p4 2	p4 3	p4 4
Analysis of the *CAD* gene only, in 3 data partions							
An albertoi	1.0	1.0	1.0	1.0	1.0	1.0	1.0
An albitarsis	1.0	1.0	1.0	1.0	1.0	1.0	1.0
An antunesi	1.0	1.0	1.0	0.998	1.0	1.0	1.0
An argyritarsis	1.0	1.0	0.954	1.0	0.980	1.0	0.972
An arthuri	1.0	1.0	1.0	1.0	1.0	1.0	1.0
An benarrochi	1.0	1.0	1.0	1.0	1.0	1.0	1.0
An braziliensis	1.0	1.0	1.0	1.0	1.0	1.0	1.0
An darlingi	1.0	1.0	1.0	1.0	1.0	1.0	1.0
An deaneorum	1.0	1.0	1.0	1.0	1.0	1.0	1.0
An dunhami	1.0	1.0	1.0	1.0	1.0	1.0	1.0
An evansae	1.0	1.0	1.0	1.0	1.0	1.0	1.0
An galvaoi	1.0	1.0	1.0	1.0	1.0	1.0	1.0
An goeldii	0.0	0.520	0.026	1.0	0.928	0.010	0.004
An konderi ss	0.972	1.0	0.870	0.974	0.922	0.984	0.960
An konderi A	0.964	0.992	0.992	0.984	0.972	0.924	0.844
An lanei	1.0	1.0	1.0	1.0	1.0	1.0	1.0
An lutzii B	0.076	0.084	0.130	0.118	0.116	0.122	0.124
An lutzii A	1.0	1.0	1.0	1.0	1.0	1.0	1.0
An lutzii ss	1.0	1.0	1.0	1.0	1.0	1.0	1.0
An marajoara	1.0	1.0	1.0	1.0	1.0	1.0	1.0
An nuneztovari	0.080	0.048	0.044	0.004	0.080	0.036	0.046
An oswaldoi A	0.084	0.036	0.172	0.122	0.138	0.108	0.128
An oswaldoi ss	0.732	0.816	0.664	0.798	0.656	0.792	0.552
An parvus	1.0	1.0	1.0	1.0	1.0	1.0	1.0
An pristinus	1.0	1.0	1.0	0.996	1.0	1.0	1.0
An rangeli	1.0	1.0	1.0	1.0	1.0	1.0	1.0
An rondoni	1.0	1.0	1.0	1.0	0.998	1.0	1.0
An strodei	0.852	0.976	0.776	0.862	1.0	0.864	1.0
An strodei CPform	1.0	1.0	1.0	1.0	1.0	1.0	1.0
An triannulatus	1.0	1.0	0.968	1.0	0.914	1.0	0.866
Analysis of the *COI* gene only, in 2 data partions							
An albertoi	0.0	0.0	0.0	0.0	0.0	0.0	0.0
An albitarsis	0.976	0.988	0.994	0.992	0.992	0.996	0.980
An antunesi	0.476	0.496	0.500	0.538	0.450	0.486	0.472
An argyritarsis	1.0	1.0	1.0	1.0	1.0	1.0	1.0
An arthuri	0.172	0.208	0.208	0.210	0.222	0.192	0.210
An atacamensis	1.0	0.996	0.998	0.994	0.998	0.996	1.0
An benarrochi	1.0	1.0	1.0	1.0	1.0	1.0	1.0
An braziliensis	1.0	1.0	1.0	1.0	1.0	1.0	1.0
An darlingi	1.0	1.0	1.0	1.0	1.0	1.0	1.0
An deaneorum	0.932	0.936	0.958	0.956	0.964	0.958	0.978
An dunhami	0.228	0.236	0.308	0.256	0.330	0.270	0.270
An evansae	0.0	0.0	0.0	0.0	0.0	0.0	0.0
An galvaoi	1.0	1.0	1.0	1.0	1.0	1.0	1.0
An goeldii	0.0	0.0	0.0	0.0	0.0	0.0	0.0
An guarani	1.0	1.0	1.0	1.0	1.0	1.0	1.0
An konderi ss	0.532	0.540	0.620	0.424	0.474	0.372	0.524
An konderi A	0.996	0.992	0.992	1.0	0.992	0.998	0.994
An lutzii B	0.024	0.016	0.028	0.034	0.032	0.038	0.028
An lutzii A	1.0	1.0	1.0	1.0	1.0	1.0	1.0
An lutzii ss	1.0	1.0	1.0	1.0	1.0	1.0	1.0
An marajoara	0.956	0.940	0.952	0.920	0.944	0.956	0.928
An nuneztovari	0.0	0.0	0.0	0.0	0.0	0.0	0.0
An oswaldoi A	1.0	1.0	1.0	1.0	1.0	1.0	1.0
An oswaldoi ss	0.0	0.0	0.0	0.0	0.0	0.0	0.0
An parvus	1.0	1.0	1.0	1.0	1.0	1.0	1.0
An pristinus	1.0	1.0	1.0	1.0	1.0	1.0	1.0
An rangeli	1.0	1.0	1.0	1.0	1.0	1.0	1.0
An rondoni	0.700	0.640	0.678	0.648	0.638	0.680	0.644
An strodei	0.0	0.004	0.0	0.0	0.002	0.0	0.004
An strodei CPform	1.0	1.0	1.0	1.0	1.0	1.0	1.0
An triannulatus	1.0	1.0	1.0	1.0	1.0	1.0	1.0
