# Supplementary material for: Phylogenetic Analysis and DNA-based Species Confirmation in Anopheles (Nyssorhynchus)
Source: PLoS One. 2013 Feb 4;8(2):e54063. doi: 10.1371/journal.pone.0054063 (PMC3563636; doi:10.1371/journal.pone.0054063)
Supplement: Table S5 — Intraspecifc K2P distances greater than 3%: Possible candidates for new species. One of the pair would be a possible candidate for placement in another species. (PDF) [file pone.0054063.s007.pdf]

Supplemental Table S5: Intraspecifc K2P distances greater than 3%: Possible candidates for new species

This table shows pairwise intraspecific K2P distances that exceed 3%. One of the pair would be a possible candidate for placement in another species.

K2P distance		
0.0357	An_parvus_AS5_1	An_parvus_MG07_9_1
0.0357	An_parvus_AS5_2	An_parvus_MG07_9_1
0.0329	An_parvus_AS5_3	An_parvus_MG07_9_1
0.0329	An_parvus_AS5_4	An_parvus_MG07_9_1
0.0362	An_parvus_MG07_9_1	An_parvus_MG56_2
0.0346	An_parvus_MG07_9_1	An_parvus_PR28_18_1
0.0345	An_parvus_MG07_9_1	An_parvus_PR28_5_1
0.0362	An_parvus_MG07_9_1	An_parvus_PR28_65_6
0.0411	An_argyritarsis_CE17_14_100	An_argyritarsis_sl_MG25_4
0.0408	An_argyritarsis_CE20_18_A	An_argyritarsis_sl_MG25_4
0.0392	An_argyritarsis_CE20_8_3	An_argyritarsis_sl_MG25_4
0.0424	An_argyritarsis_MG04_03	An_argyritarsis_sl_MG25_4
0.0334	An_triannulatus_AC1_108	An_triannulatus_ES03_03_01
0.0301	An_triannulatus_ES03_03_01	An_triannulatus_SP09_02
0.0343	An_oswaldoi_ES08_11_07	An_oswaldoi_SP22_9
0.0360	An_oswaldoi_SP03_06	An_oswaldoi_SP22_9
0.0492	An_lutzii_B369	An_lutzii_sl1_RS16a
0.0492	An_lutzii_B369	An_lutzii_sl1_RS16b
0.0346	An_evansae_PR19_10_104	An_evansae_SP12_44
0.0314	An_evansae_SP12_28	An_evansae_SP12_44
0.0346	An_evansae_SP12_44	An_evansae_SP18_27
0.0330	An_evansae_SP12_44	An_evansae_VP06_7_4
