# Supplementary material for: Phylogenetic Analysis and DNA-based Species Confirmation in Anopheles (Nyssorhynchus)
Source: PLoS One. 2013 Feb 4;8(2):e54063. doi: 10.1371/journal.pone.0054063 (PMC3563636; doi:10.1371/journal.pone.0054063)
Supplement: Table S7 — DNA amplification primers. (DOC) [file pone.0054063.s009.doc]

Supplementary Table S7. DNA amplification primers.

| **Gene** | **Primer** | **Sequence 5' - 3'** | Source |
| --- | --- | --- | --- |
| COI | C1-J-1718^a,b^ | GGA GGA TTT GGA AAT TGA TTA GTT CC | Simon et al. (1994) |
|  | C1-N-2191^a,b^ | CCC GGT AAA ATT AAA ATA TAA ACT TC | Simon et al. (1994) |
|  |  |  |  |
|  | LCO1490^b^ | GGT CAA CAA ATC ATA AAG ATA TTG G | Folmer et al. (1994) |
|  | HCO2198^b^ | TAA ACT TCA GGG TGA CCA AAA AAT CA | Folmer et al. (1994) |
|  |  |  |  |
| CAD | CAD338F^c^ | ATG AAR TAY GGY AAT CGT GGH CAY AA | Moulton and Wiegmann (2004) |
|  | CAD680R^c^ | AAN GCR TCN CGN ACM ACY TCR TAY TC | Moulton and Wiegmann (2004) |
|  | CAD_F^d^ | CCM RSC GST GCT ACA TGA C | This study |
|  | CAD_R^d^ | GAT GAT GAG CTG RGY CGA GTG | This study |
|  | CAD338F_M13^c^ | TGT AAA ACG ACG GCC AGT ATG AAR TAY GGY AAT CGT GGH CAY AA | This study |
|  | CAD680R_M13^c^ | CAG GAA ACA GCT ATG ACC AAN GCR TCN CGN ACM ACY TCR TAY TC | This study |
|  |  |  |  |
| White | WZ2E^c^ | AAY TAY AAY CCI GCI GAY TTY TA | Zwiebel et al. (1995) |
|  | WZ11X^c^ | TTI ARR AAR AAI CCI CCR AA | Zwiebel et al. (1995) |
|  | W1F^d^ | GAT CAA RAA GAT CTG YGA CTC GTT | Bourke et al. (2010) |
|  | W2R^d^ | GCC ATC GAG ATG GAG GAG CTG | Bourke et al. (2010) |
|  |  |  |  |
| - | M13F^e^ | TGT AAA ACG ACG GCC AGT | - |
| - | M13R^e^ | CAG GAA ACA GCT ATG ACC | - |

^a^Used only for GQ902966 and GQ902967**. ^b^**Used for PCR and sequencing reactions.^c^Used for PCR only. ^d^Internal primers used for sequencing reactions. ^e^Used for sequencing reactions of PCR products generated with CAD338F_M13 and CAD680R_M13.
