# Supplementary material for: Phylogenetic Analysis and DNA-based Species Confirmation in Anopheles (Nyssorhynchus)
Source: PLoS One. 2013 Feb 4;8(2):e54063. doi: 10.1371/journal.pone.0054063 (PMC3563636; doi:10.1371/journal.pone.0054063)
Supplement: Table S8 — Specimens and sequences used. A table showing which sequences were used in the analysis, and what name was given to those sequences. (PDF) [file pone.0054063.s010.pdf]

Supplemental Table S8. Specimens and sequences used

Three genes were used — the nuclear *white* and *CAD* genes, and the mitochondrial *COI* gene. There were, in the sequences from the three genes, 144 specimens represented.

	Name	White	CAD	COI
1	An_albertoi_MG07_12_4	+	+	+
2	An_albertoi_MG07_7_10	+	+	+
3	An_albitarsis_MG11_20_3	+	+	+
4	An_albitarsis_SP104_2_2	+	+	+
5	An_albitarsis_VP06_01_01	+	+	+
6	An_antunesi_RJ03_11	+	+	+
7	An_antunesi_RJ03_12	+	+	+
8	An_antunesi_RJ03_13	+	+	+
9	An_antunesi_RJ03_6	+	+	+
10	An_antunesi_VP09_17	+	+	+
11	An_antunesi_VP11b	+		+
12	An_argyritarsis_CE17_14_100	+	+	+
13	An_argyritarsis_CE20_18_A	+	+	+
14	An_argyritarsis_CE20_8_3	+	+	+
15	An_argyritarsis_MG04_03	+	+	+
16	An_argyritarsis_sl_MG25_4	+	+	+
17	An_arthuri_MG07_6_3	+	+	+
18	An_arthuri_MG24_1	+	+	+
19	An_arthuri_RO08_104	+	+	+
20	An_arthuri_RO8_1	+	+	+
21	An_arthuri_SP31_120	+	+	+
22	An_atacamensis_GQ902966			+
23	An_atacamensis_GQ902967			+
24	An_atacamensis_JF923686			+
25	An_benarrochi_AC15_109	+	+	+
26	An_benarrochi_AC18_115	+	+	+
27	An_benarrochi_AC18_117	+	+	+
28	An_benarrochi_AC18_120	+	+	+
29	An_braziliensis_AP21_39_3	+	+	+
30	An_braziliensis_SP16_03	+	+	+
31	An_cruzii_ST16	+	+	+
32	An_darlingi_AC20_21_100	+	+	+
33	An_darlingi_AP13_03_06	+	+	+
34	An_darlingi_AP17_01_10	+	+	+
35	An_deaneorum_AC01_07	+	+	+
36	An_deaneorum_AC02_02	+	+	+
37	An_deaneorum_MS08_127	+	+	+
38	An_dunhami_BRAM13_06	+	+	+
39	An_dunhami_BRAM13_07	+	+	+
40	An_dunhami_BRAM13_113	+	+	+
41	An_evansae_PR19_10_104	+	+	+
42	An_evansae_SP12_28	+	+	+
43	An_evansae_SP12_44	+	+	+
44	An_evansae_SP18_106	+	+	+
45	An_evansae_SP18_27	+		+
46	An_evansae_VP06_7_4	+	+	+
47	An_galvaoi_PR19_2_101	+	+	+
48	An_galvaoi_SP18_111	+	+	+
49	An_galvaoi_SP66_20_1	+		+
50	An_goeldii_BRAM03_01	+		+
51	An_goeldii_BRAM22_101	+	+	+
52	An_goeldii_PA7_02_02	+	+	+
53	An_goeldii_PA7_03_08	+	+	+
54	An_goeldii_PA7_04_03	+	+	+
55	An_goeldii_PA7_17_02	+		+
56	An_guarani_PR29	+	+	+
57	An_guarani_PR29_08			+
58	An_guarani_PR29_09_06			+
59	An_intermedius_SP22_106	+	+	+
60	An_kompi_SP69_22_5	+		+
61	An_konderi_AC18_16	+	+	+
62	An_konderi_AP15_11	+	+	+
63	An_konderi_AP21_43	+	+	+
64	An_konderi_AP25_11_24	+	+	+
65	An_konderi_AP25_1_100	+	+	+
66	An_konderi_PR06_2_13	+	+	+
67	An_konderi_PR14_1_9	+	+	+
68	An_konderi_PR14_3_17	+	+	+
69	An_konderi_PR14_9_108	+	+	+
70	An_konderi_RO18_1_6	+	+	+
71	An_lanei_CJ02_02	+	+	+
72	An_lanei_CJ02_03	+	+	+
73	An_lutzii_A325	+	+	+
74	An_lutzii_B369	+	+	+
75	An_lutzii_SP02_10_5	+		+
76	An_lutzii_SP02_11_9	+	+	+
77	An_lutzii_SP02_12_1	+	+	+
78	An_lutzii_SP02_13_3	+	+	+
79	An_lutzii_SP02_14_6	+	+	+
80	An_lutzii_SP02_15_5	+	+	+
81	An_lutzii_SP02_9_2	+	+	+
82	An_lutzii_sl1_RS16a	+	+	+
83	An_lutzii_sl1_RS16b	+	+	+
84	An_lutzii_sl2_RS19_13	+	+	+
85	An_lutzii_sl2_RS19_21	+	+	+
86	An_lutzii_sl2_RS19_22	+	+	+
87	An_lutzii_sl2_RS33_105	+	+	+
88	An_lutzii_sl2_RS33_3	+	+	+
89	An_lutzii_sl2_RS33a	+	+	+
90	An_lutzii_sl2_RS33b	+	+	+
91	An_marajoara_AP21_50_1	+	+	+
92	An_marajoara_AP5_01_04	+	+	+
93	An_marajoara_PA3_1_13	+	+	+
94	An_nuneztovari_RO1_107	+	+	+
95	An_nuneztovari_RO20_02_03	+	+	+
96	An_nuneztovari_RO2_13	+	+	+
97	An_nuneztovari_RO4_02	+	+	+
98	An_oryzalimnetes_SP09_03	+	+	+
99	An_oswaldoi_ES08_11_07	+		+
100	An_oswaldoi_SP03_06	+	+	+
101	An_oswaldoi_SP22_9	+	+	+
102	An_oswaldoi_sl_AC18_102	+	+	+
103	An_oswaldoi_sl_AC18_107	+	+	+
104	An_oswaldoi_sl_PA_15_C1F2	+	+	+
105	An_oswaldoi_sl_PA_15_C2F4		+	+
106	An_oswaldoi_sl_PA_15_C2F7		+	+
107	An_parvus_AS5_1	+	+	+
108	An_parvus_AS5_2	+	+	+
109	An_parvus_AS5_3	+	+	+
110	An_parvus_AS5_4	+	+	+
111	An_parvus_MG07_9_1	+	+	+
112	An_parvus_MG56_2	+	+	+
113	An_parvus_PR28_18_1	+	+	+
114	An_parvus_PR28_5_1	+	+	+
115	An_parvus_PR28_65_6	+	+	+
116	An_pristinus_SP50a	+	+	+
117	An_pristinus_SP50b	+	+	+
118	An_pristinus_SP51_100	+	+	+
119	An_pristinus_SP53_100	+	+	+
120	An_pristinus_SP53_101	+	+	+
121	An_pristinus_SP53_4	+	+	+
122	An_pristinus_SP53_5	+	+	+
123	An_pristinus_SP55_2	+	+	+
124	An_pristinus_SP55_4	+	+	+
125	An_pristinus_VP11a	+		+
126	An_rangeli_AC15_04	+		+
127	An_rangeli_AC18_110	+	+	+
128	An_rangeli_RO18_8_2	+	+	+
129	An_rondoni_PR28_34_100	+	+	+
130	An_rondoni_PR28_36_02	+	+	+
131	An_rondoni_PR28_55_100	+	+	+
132	An_strodei_CPform_ES20_4_1	+	+	+
133	An_strodei_CPform_MG15_01_01	+	+	+
134	An_strodei_CPform_MG15_06_12	+	+	+
135	An_strodei_CPform_PR21_110	+	+	+
136	An_strodei_ES09_1	+	+	+
137	An_strodei_MG30_102	+	+	+
138	An_strodei_SPR04_07	+	+	+
139	An_strodei_VP06_05_01	+	+	+
140	An_triannulatus_AC1_108	+	+	+
141	An_triannulatus_AP17_04_01	+	+	+
142	An_triannulatus_ES03_03_01	+	+	+
143	An_triannulatus_MG56_12_03	+	+	+
144	An_triannulatus_SP09_02	+	+	+
