# Supplementary material for: Phylogenetic Analysis and DNA-based Species Confirmation in Anopheles (Nyssorhynchus)
Source: PLoS One. 2013 Feb 4;8(2):e54063. doi: 10.1371/journal.pone.0054063 (PMC3563636; doi:10.1371/journal.pone.0054063)
Supplement: Table S9 — Model choice. Tables showing the models suggested by Modeltest and the models that were used in the model-based DNA analyses. The Akaike weights were often low, showing ambiguity in the model choice. (PDF) [file pone.0054063.s011.pdf]

## Supplementary Table S9. Model Choice

Model choice was guided by Modeltest. The choice suggested by Modeltest is shown, but as that model was often not available in the software that was used to do the analysis the model that was actually used is also shown. The AIC weights are shown in parentheses; these are often low, indicating ambiguity in the model choice.

Table S9A. For model choice for DNA with the outgroup, with *An kompi*, all three outgroup sequences were included.

| | modeltest | used |
|---|---|---|
| white143_1 | TVM (0.094) | HKY (0.091) |
| white143_2 | K80 (0.19) | K80 (0.19) |
| white143_3 | TVM+G (0.40) | GTR+G (0.19) |
| cad143_1 | TIM+G (0.28) | GTR+G (0.05) |
| cad143_2 | F81 (0.17) | F81 (0.17) |
| cad143_3 | TVM+G (0.23) | HKY+G (0.19) |
| coi143_1 | TrN (0.26) | HKY (0.04) |
| coi143_3 | GTR+G (0.70) | GTR+G (0.70) |

Table S9B. For model choice for DNA, with the outgroup but without *An kompi*, two outgroup sequences were included.

| gene | modeltest | used |
|---|---|---|
| white1 | HKY (0.16) | HKY (0.16) |
| white2 | TrNef (0.28) | K2P (0.03) |
| white3 | TVM+G (0.35) | GTR+G (0.20) |
| cad1 | TIM+G (0.32) | GTR+G (0.05) |
| cad2 | F81 (0.15) | F81 (0.015) |
| cad3 | TVM+G (0.23) | HKY+G (0.19) |
| coi1 | TrN (0.20) | HKY (0.10) |
| coi3 | GTR+G (0.69) | GTR+G (0.69) |
